# ﻿Didymellaceae species associated with tea plant (*Camelliasinensis*) in China

**DOI:** 10.3897/mycokeys.105.119536

**Published:** 2024-05-29

**Authors:** Yuchun Wang, Yiyi Tu, Xueling Chen, Hong Jiang, Hengze Ren, Qinhua Lu, Chaoling Wei, Wuyun Lv

**Affiliations:** 1 College of Tea Science and Tea Culture, Zhejiang A & F University, Hangzhou 311300, Zhejiang, China Zhejiang A & F University Hangzhou China; 2 Institute of Sericulture and Tea, Zhejiang Academy of Agricultural Sciences, Hangzhou 310021, China Institute of Sericulture and Tea, Zhejiang Academy of Agricultural Sciences Hangzhou China; 3 State Key Laboratory of Tea Plant Biology and Utilization, Anhui Agricultural University, 130 Changjiang West Road, Hefei, 230036, Anhui, China Anhui Agricultural University Hefei China

**Keywords:** *Camellia* inhibiting fungi, *Didymella*, distribution, *Epicoccum*, leaf blight, *Neoascochyta*, new species, pathogenicity

## Abstract

Tea plant is one of the most important commercial crops worldwide. The Didymellaceae fungi can cause leaf blight disease of tea plant. In this study, 240 isolates were isolated from tea plant leaves of 10 provinces in China. Combined with multi-locus (ITS, LSU, *RPB2* and *TUB2*) phylogenetic analysis and morphological characteristics, these isolates were identified as 25 species of six genera in Didymellaceae, including 19 known species *Didymellacoffeae-arabicae*, *D.pomorum*, *D.segeticola*, *D.sinensis*, *Epicoccumcatenisporum*, *E.dendrobii*, *E.draconis*, *E.italicum*, *E.latusicollum*, *E.mackenziei*, *E.oryzae*, *E.poaceicola*, *E.rosae*, *E.sorghinum*, *E.tobaicum*, *Neoascochytamortariensis*, *Paraboeremialitseae*, *Remotididymellaanemophila* and *Stagonosporopsiscaricae*, of which 15 species were new record species and six novel species, named *D.yunnanensis*, *E.anhuiense*, *E.jingdongense*, *E.puerense*, *N.yunnanensis* and *N.zhejiangensis*. Amongst all isolates, *D.segeticola* was the most dominant species. Pathogenicity tests on tea plant leaves showed that *E.anhuiense* had the strongest virulence, while *E.puerense* had the weakest virulence. Besides, *D.pomorum*, *D.yunnanensis*, *E.dendrobii*, *E.italicum*, *E.jingdongense*, *E.mackenziei*, *E.oryzae*, *E.rosae*, *E.tobaicum*, *N.mortariensis*, *N.yunnanensis*, *N.zhejiangensis* and *R.anemophila* were non-pathogenic to the tea plant.

## ﻿Introduction

*Pleosporales* is a predominant order with a worldwide distribution in terrestrial and aquatic environments (An et al. 2022). In these environments, *Pleosporales* mainly survives as saprophytic fungi on dead leaves or stems (Kodsueb et al. 2006; Zhang et al. 2009a, 2009b). It also can be endophytes, epiphytes and parasites of green leaves or stems and lichens (Calatayud et al. 2001; Kruys et al. 2006; Huang et al. 2008). Didymellaceae is one of the largest family in *Pleosporales*, which was established by de Gruyter et al. (2009). It is widely distributed geographically, existing in different ecosystems, such as air, soil, water, house dust and coral and parasitising in other fungi and lichens (Sutton 1980; Chen et al. 2017; Wanasinghe et al. 2018a). Previous studies have reported that this family included three main genera: *Ascochyta*, *Didymella* and *Phoma*, as well as other allied phoma-like genera which grouped in the Didymellaceae (Chen et al. 2017). Besides, *Leptosphaerulina* and *Macroventura* were genetically closely similar and classified into Didymellaceae (Silva-Hanlin and Hanlin 1999; Kodsueb et al. 2006; Aveskamp et al. 2010). Aveskamp et al. (2010) divided the family into at least 18 different clusters according to the sequence data obtained from 324 strains, redefining *Epicoccum*, *Peyronellaea* and *Stagonosporopsis* and demonstrating that *Ascochyta*, *Phoma* and *Didymella* were highly polyphyletic. As an extremely species-rich family, more than 5400 species belonging to 44 accepted genera have been recorded in Didymellaceae (Kularathnage et al. 2023).

Although the basic taxonomy of Didymellaceae has been established, the problem of multi-source of many genera has not been solved. Morphological characteristics, coupled with multi-gene molecular phylogeny, have developed as a more effective strategy for the identification of Didymellaceae, which has improved the understanding of the taxonomy (Hou et al. 2020a). For example, combining morphological observation and multi-locus phylogenetic analysis, based on ITS (the internal transcribed spacer region of the rDNA gene), LSU (partial large subunit nrDNA nucleotide sequences), *RPB2* (the RNA polymerase II second largest subunit gene) and *TUB2* (partial gene regions of β-tubulin), Chen et al. (2015a) clarified the generic delimitation in Didymellaceae. Seventeen fully-supported monophyletic branches in Didymellaceae were revealed and the generic circumscriptions of *Ascochyta*, *Phoma* and *Didymella* emended. Recently, 108 Didymellaceae isolates newly obtained from 40 host plant species in 27 plant families in China and other countries were investigated (Chen et al. 2017). Amongst these, 68 isolates representing 32 new taxa are recognised, based on morphological differences and the multi-locus phylogeny using sequences of ITS, LSU, *RPB2* and *TUB2* and a total of 19 genera are recognised in the Didymellaceae family (Chen et al. 2017). Wanasinghe et al. (2018a) isolated didymellaceous taxa from *Alhagipseudalhagi*, *Coronillaemerus*, *Cytisus* sp., *Elaeagnusangustifolia* and *Spartiumjunceum* in Italy, Russia and Uzbekistan and present comprehensive morphological descriptions and in-depth phylogenetic investigation of five new species, including *Ascochytacoronillae-emeri*, *Microsphaeropsisspartii-juncei*, *Neomicrosphaeropsisalhagi-pseudalhagi*, *N.cytisicola* and *N.elaeagni*. Furthermore, as a cosmopolitan family, 1124 Didymellaceae strains globally collected from 92 countries, 121 plant families and 55 other substrates were examined via multi-locus phylogenetic analyses and detailed morphological comparisons (Hou et al. 2020b). Seven new genera, including *Dimorphoma*, *Ectodidymella*, *Longididymella*, *Macroascochyta*, *Paramicrosphaeropsis*, *Pseudopeyronellaea* and *Sclerotiophoma* were newly introduced in Didymellaceae (Hou et al. 2020b). In addition, 40 new species were identified combining phylogenetic analyses, based on concatenated DNA sequence dataset (ITS, LSU, *RPB2* and *TUB2*) and morphological examination (Hou et al. 2020b). Given the above, phylogenetic analyses, based on a combined ITS-LSU- *RPB2*-*TUB2* DNA sequence dataset, have been demonstrated as an effective method for the identification of Didymellaceae at species level (Hou et al. 2020a; Yuan et al. 2021; Kularathnage et al. 2023; Yang et al. 2023a).

Tea plant (*Camelliasinensis*) is one of the important commercial crops, which is widely cultivated in tropical and subtropical areas (Manawasinghe et al. 2021). Leaf blight disease caused by phytopathogens from Didymellaceae threatens the healthy growth of tea plants (Chen et al. 2017; Manawasinghe et al. 2021; Kumhar et al. 2022; Huang et al. 2023). Some species of Didymellaceae, such as *Didymellasegeticola*, *D.bellidis*, *Epicoccumcamelliae*, *E.latusicollum*, *E.layuense* and *E.sorghinum*, were isolated from diseased tissues (Chen et al. 2017; Ren et al. 2019; Manawasinghe et al. 2021; Wang et al. 2021). However, comprehensive understanding on the biodiversity and pathogenicity of Didymellaceae on tea plants remains unknown. Thus, to systematically and comprehensively elaborate the species of Didymellaceae in tea plant can provide further insight into the understanding of pathogens causing leaf blight disease.

In this study, 240 isolates of Didymellaceae were obtained from tea plant leaves of ten provinces in China. We aimed to clarify the classification of these isolates using phylogenetic analyses, based on the multi-locus (ITS, LSU, *RPB2* and *TUB2*) DNA sequences and, thus, determined the biodiversity of Didymellaceae on tea plants. In addition, to evaluate the pathogenicity of isolates, we performed pathogenicity tests with 36 representative isolates on leaves of *C.sinensis* cv. *Longjing43* (LJ43), a relative susceptible cultivar (Wang et al. 2016). The pathogenicity results will preliminarily determine the dominant species associated with leaf blight.

## ﻿Materials and methods

### ﻿Collection and isolates

**Figure 1.**
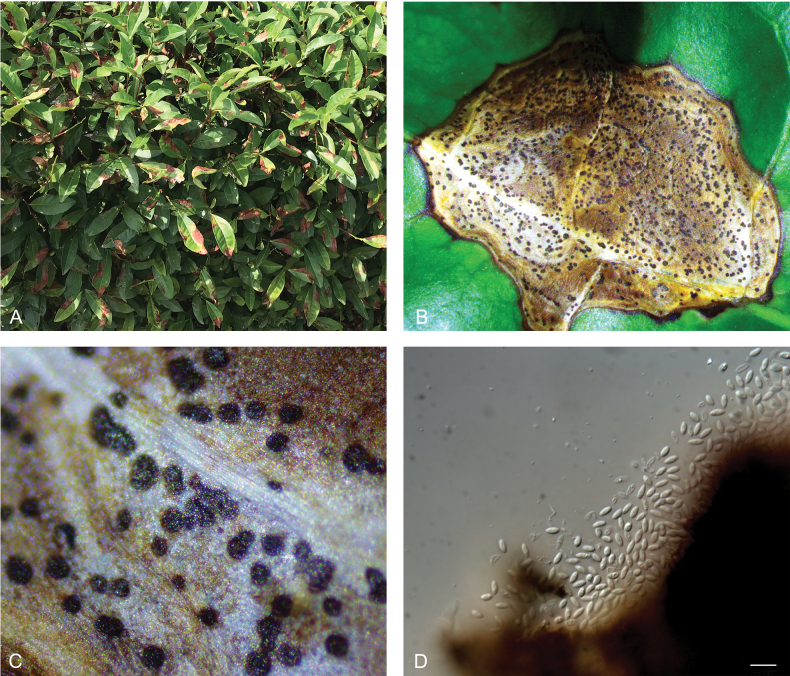
Disease symptoms on *Camelliasinensis* caused by Didymellaceae**A** leaf symptom **B** fungal fruitbody structures formed on leaves **C** close-up of fungal fruitbody structures **D** conidia. Scale bars: 10 μm.

The isolates were collected from tea plants in 15 cities of ten provinces in China, including Hangzhou (30°18'N, 120°09'E), Lishui (28°66'N, 120°09'E) and Shaoxing (30°08'N, 120°49'E) Cities in Zhejiang Province, Huangshan (29°72'N, 118°32'E) and Anqing (30°69'N, 116°40'E) Cities in Anhui Province, Yixing (31°28'N, 119°72'E) and Wuxi (31°47'N, 120°27'E) Cities in Jiangsu Province, Chengdu (30°24'N, 103°51'E) and Guangyuan (32°64'N, 105°89'E) Cities in Sichuan Province, Wuhan (30°30'N, 114°14'E) City in Hubei Province, Nanchang (28°55'N, 115°94'E) City in Jiangxi Province, Tongren (27°96'N, 109°28'E) City in Guizhou Province, Xinyang (32°12'N, 114°06'E) City in Henan Province, Yingde City (39°91'N, 116°52'E) in Guangdong Province and Puer (24°45'N, 100°83'E) City in Yunnan Province. The fungal strains were obtained by two different methods, one was tissue isolation from healthy leaves and the other was single spore isolation by scraping diseased spots from diseased leaves (Fig. 1) (Cai et al. 2009; Wang et al. 2016). For single spore isolation, spores were isolated from diseased leaves and suspended in sterilised ddH_2_O under sterilised conditions, then were coated on potato dextrose agar (PDA) plates and cultured at 25 °C in the dark. For tissue isolation, healthy leaves were surface-sterilised and then cultured on PDA plates at 25 °C in the dark. After 2 days, single colonies were selected and transferred to new PDA plates for further pure cultivation. For further study, pure cultures were stored in 25% glycerol at -80 °C.

Type specimens of new species from this study were deposited in the Mycological Herbarium, Institute of Microbiology, Chinese Academy of Sciences, Beijing, China (HMAS) and ex-type living cultures were deposited in the China General Microbiological Culture Collection Center (CGMCC). The descriptions of the novel species reported in this study were submitted to the MycoBank database (https://www.mycobank.org).

### ﻿DNA extraction, PCR amplification and sequencing

Isolates were cultured at 28 °C in the dark for 7 days. Genomic DNA was extracted from fresh mycelia using Genomic DNA Purification Kit (Sangon Biotechnology (Shanghai) Co., Ltd., China). The fragments of ITS, LSU, *RPB2* and *TUB2* were amplified by PCR using the genomic DNA as the template (Chen et al. 2015a). PCR amplifications were performed in a reaction mixture consisting of 12 μl 2× Taq Master Mix, 1 μl 10 μM forward primer, 1 μl 10 μM reverse primer, 1 μl DNA template, adjusted to a final volume of 25 μl with ddH_2_O. Primer pairs used in this study were listed in Table 1. The PCR amplification procedures of four loci were as follows: ITS, predenaturation at 95 °C for 5 min, followed by 35 cycles of denaturation at 95 °C for 30 s, annealing at 48 °C for 30 s and extension at 72 °C for 2 min, with the final extension at 72 °C for 10 min; LSU, predenaturation at 95 °C for 5 min, followed by 35 cycles of denaturation at 95 °C for 45 s, annealing at 48 °C for 45 s and extension at 72 °C for 2 min, with the final extension at 72 °C for 10 min; *RPB2*, predenaturation at 94 °C for 5 min, followed by 35 cycles of denaturation at 95 °C for 45 s, annealing at 56 °C for 80 s and extension at 72 °C for 2 min, with the final extension at 72 °C for 10 min; *TUB2*, predenaturation at 95 °C for 5 min, followed by 35 cycles of denaturation at 95 °C for 30 s, annealing at 52 °C for 30 s and extension at 72 °C for 80 s, with the final extension at 72 °C for 10 min. The PCR products were visualised using 1% agarose electrophoresis gels. Sequencing was performed by Youkang Biotechnology (Hangzhou) Co., Ltd., China.

**Table 1.** Primer pairs used in this study.

Gene	Primer	Primer sequence (5’-3’)
ITS	ITS5	GGAAGTAAAAGTCGTAACAAGG
	ITS4	TCCTCCGCTTATTGATATGC
LSU	LROR	GTACCCGCTGAACTTAAGC
	LR7	TACTACCACCAAGATCT
*RPB2*	RPB2-5f2	GGGGWGAYCAGAAGAAGGC
	RPB2-7cR	CCCATRGCTTGYTTRCCCAT
*TUB2*	Btub2Fd	GTBCACCTYCARACCGGYCARTG
	Btub4Rd	CCRGAYTGRCCRAARACRAAGTTGTC

### ﻿Phylogenetic analysis

Sequences of the ITS, LSU, *RPB2* and *TUB2* loci for all the isolates were blasted against the National Center for Biotechnology Information (NCBI) GenBank nucleotide datasets (http://www.ncbi.nlm.nih.gov/Blast.cgi) (Suppl. material 1). Alignments of ITS, LSU, *RPB2* and *TUB2* sequences were generated with MAFFT v.7.525 (Katoh et al. 2019) and MEGA v.6.0 software was used for manual correction (Tamura et al. 2013). To investigate the phylogenetic relationships between different isolates, both Bayesian Inference (BI) and Maximum Likelihood (ML) methods were used and followed by the concatenated alignments (Han et al. 2023). For BI analysis, Markov Chain Monte Carlo (MCMC) sampling was used to reconstruct phylogenies in MrBayes v.3.2 (Ronquist and Huelsenbeck 2003). For ML analysis, the substitution model (GTR + I + G model with gamma-distributed rate) were selected (Wang et al. 2016). Phylograms were created in FigTree v. 1.3.1 (Rambaut and Drummond 2008) and edited in Adobe Illustrator 2022 (available from https://www.adobe.com/cn/creativecloud/roc/business.html).

### ﻿Morphology

Isolates were grown on oatmeal agar (OA) and PDA plates and cultured at 28 °C for 7 days (Hou et al. 2020b). Colony diameters of each strain with three replicates were then measured and repeated at least three times. The morphological characteristics were determined after another 7 days (Boerema et al. 2004). The shape, colour and size of mature pycnidia and conidia were observed under light microscopy (SOPTOP-CX40RFL, China). Sizes of at least 30 conidia were measured with the light microscopy. The description of new species is mainly based on the morphology of colony, conidia and pycnidia, conidia size, colony growth rate and aerial hyphae on OA and PDA.

### ﻿Pathogenicity tests

Asymptomatic leaves were collected from 5-year-old LJ43 grown in a tea garden in Hangzhou, Zhejiang Province, China. The fourth leaf of current-growth branches was cut off for the analysis. The detached leaves were surface-sterilised with 75% alcohol and washed with sterilised ddH_2_O twice and air dried. A 5-mm mycelial disc cut from the edge of 7-day-old cultures was inoculated both sides of leaves after wounding with a sterilised needle (using a pattern of puncture perpendicular to the leaf to create the same number of wounds and this pattern was applied uniformly across all leaves) and cultured directly on a moist surface in the dark with 100% humidity at 28 °C for 3 days (Solarte et al. 2017). After 3 days, the lesion diameters were measured and photographed. Each strain with at least three replicates was repeated three times. Thirty-six representative isolates were selected for the pathogenicity test, including *D.pomorum* YCW196, *D.segeticola* YCW109, YCW192, YCW1135, YCW1289 and YCW2007, *D.sinensis* YCW1884 and YCW2118, *D.yunnanensis*CGMCC 3.24241 (YCW1909), *E.anhuiense* YCW961 and YCW1829, *E.dendrobii* YCW1866, *E.draconis* YCW101 and YCW187, *E.italicum* YCW2005, *E.jingdongense* YCW1868 and YCW1937, *E.latusicollum* YCW1921, *E.mackenziei* YCW1965 and YCW1967, *E.oryzae* YCW2010, *E.poaceicola* YCW1948 and YCW2115, *E.rosae* YCW331, *E.poerense* YCW224 and YCW2117, *E.tobaicum* YCW372, *Neoascochytamortariensis* YCW1346, *N.yunnanensis* YCW1883, *N.zhejiangensis* YCW1361 and YCW1107, *Paraboeremialitseae* YCW1356 and YCW1363, *Remotididymellaanemophila* YCW434 and *Stagonosporopsiscaricae* YCW1928 and YCW1977.

### ﻿Statistical analysis

The average value of all measurements was analysed using the SPSS Inc. software (IBM, New York, USA). The lesion sizes data were analysed with one-way ANOVA (analysis of variance) and the least significant difference (LSD) test and the values were presented as the mean ± SE (standard error) of three repeats. A *P* value < 0.05 was considered statistically significant according to the LSD test.

## ﻿Results

### ﻿Isolates and phylogenetic analysis

In this study, 240 isolates were obtained from tea plant leaves of ten provinces in China. A multi-locus phylogeny was constructed, based on four loci (ITS, LSU, *RPB2* and *TUB2*). The ML tree from each alignment is presented, with bootstrap support values and Bayesian posterior values plotted at each node. All isolates were recognised and clustered into six genera in Didymellaceae, including *Didymella*, *Epicoccum*, *Neoascochyta*, *Paraboeremia*, *Remotididymella* and *Stagonosporopsis*.

**Figure 2.**
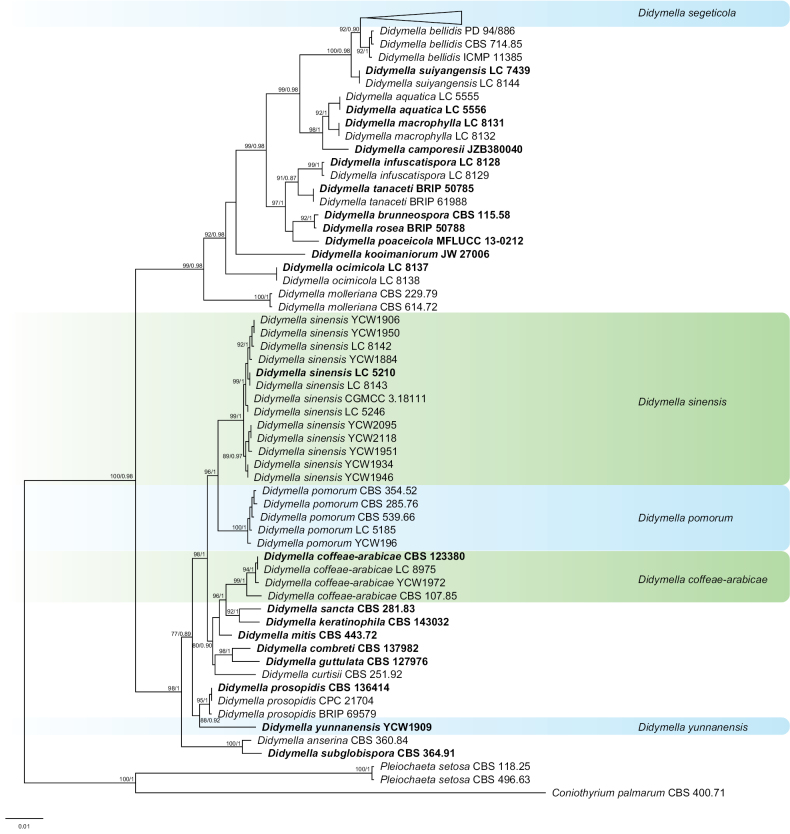
Phylogenetic tree generated by Maximum Likelihood analysis, based on the combined ITS, LSU, *RPB2* and *TUB2* dataset of *Didymella* species. Bootstrap support values above 50% and Bayesian posterior values above 0.75 are shown at each node (ML/PP). *Pleiochaetasetosa* CBS 118.25 / CBS 496.63 and *Coniothyriumpalmarum* CBS 400.71 are used as outgroups. Ex-type strains are emphasised in bold.

For *Didymella* genus, phylogenetic analysis was performed with the combined sequence data from 227 isolates, including 45 referenced strains and 182 newly-sequenced strains. The 227 isolates comprised 2453 characters (ITS = 1–540 bp, LSU = 1504–2465 bp, *RPB2* = 545–1146 bp and *TUB2* = 1151–1499 bp) after alignment. *Pleiochaetasetosa* CBS 118.25 / CBS 496.63 and *Coniothyriumpalmarum* CBS 400.71 were used as the outgroup. Of the 182 new isolates, 171 isolates clustered with *D.segeticola* and retrieved 92% ML and 0.90 PP support, eight clustered with *D.sinensis* (99% in ML and 1 in PP), one clustered with *D.pomorum* (100% in ML and 1 in PP) and one clustered with *D.coffeae-arabicae* (94% in ML and 1 in PP). One isolate formed a new clade named *D.yunnanensis* (88% in ML and 0.92 in PP), which showed a close phylogenetic affinity to *D.prosopidis* (CBS 136414, CPC 21704 and BRIP 69579) (Fig. 2).

**Figure 3.**
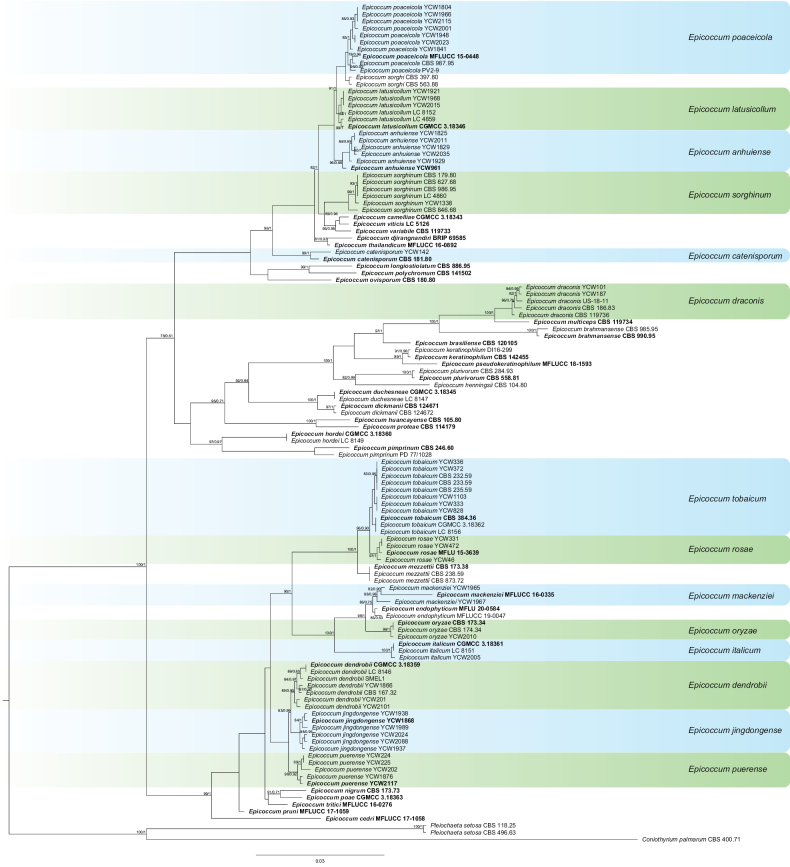
Phylogenetic tree generated by Maximum Likelihood analysis, based on the combined ITS, LSU, *RPB2* and *TUB2* dataset of *Epicoccum* species. Bootstrap support values above 50% and Bayesian posterior values above 0.75 are shown at each node (ML/PP). *Pleiochaetasetosa* CBS 118.25 / CBS 496.63 and *Co.palmarum* CBS 400.71 are used as outgroups. Ex-type strains are emphasised in bold.

For *Epicoccum* genus, phylogenetic analysis was performed with the combined sequence data from 114 isolates, including 68 referenced strains and 46 newly-sequenced strains. The 114 isolates comprised 2466 characters (ITS = 1–559 bp, LSU = 1516–2478 bp, *RPB2* = 564–1162 bp and *TUB2* = 1167–1511 bp) after alignment. *Pleiochaetasetosa* CBS 118.25 / CBS 496.63 and *Co.palmarum* CBS 400.71 were used as the outgroups. Of the 46 new isolates, seven isolates clustered with *E.poaceicola* (78% in ML and 0.96 in PP), three clustered with *E.latusicollum* (84% in ML and 1 in PP), one clustered with *E.sorghinum* (99% in ML and 1 in PP), one clustered with *E.catenisporum* (99% in ML and 1 in PP), three clustered with *E.dendrobii* (89% in ML and 0.95 in PP), two clustered with *E.draconis* (96% in ML and 0.76 in PP), five clustered with *E.tobaicum* (96% in ML and 0.90 in PP), three clustered with *E.rosae* (97% in ML and 1 in PP), two clustered with *E.mackenziei* (88% in ML and 0.98 in PP), one clustered with *E.oryzae* (99% in ML and 1 in PP), one clustered with *E.italicum* (100% in ML and 1 in PP) and 17 unidentified isolates did not match any known lineage of *Epicoccum* species. Amongst the 17 unidentified isolates, six isolates formed a new monophyletic clade named *E.anhuiense* with support values 96% in ML and 0.68 in PP, six formed a new clade named *E.jingdongense* showing a close phylogenetic affinity to *E.dendrobii* in the combined phylogeny with 83% ML and 0.99 PP support and five formed a new monophyletic clade named *E.puerense* with high support (98% in ML and 0.92 in PP) (Fig. 3).

**Figure 4.**
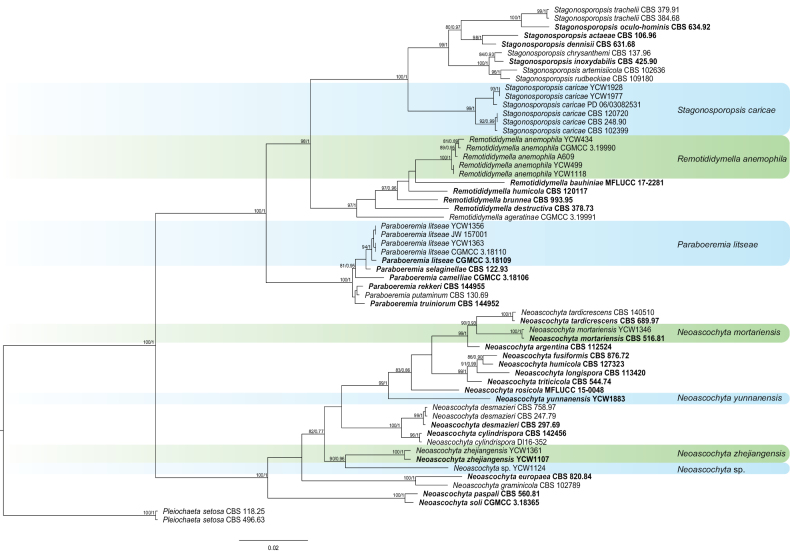
Phylogenetic tree generated by Maximum Likelihood analysis, based on the combined ITS, LSU, *RPB2* and *TUB2* dataset of *Neoascochyta*, *Paraboeremia*, *Remotididymella* and *Stagonosporopsis* species. Bootstrap support values above 50% and Bayesian posterior values above 0.75 are shown at each node (ML/PP). *Pleiochaetasetosa* CBS 118.25 / CBS 496.63 is used as the outgroup. Ex-type strains are emphasised in bold.

For other genera, phylogenetic analysis was performed with the combined sequence data from 56 isolates, including 44 referenced strains and 12 newly-sequenced strains. The 56 isolates comprised 2385 characters (ITS = 1–480 bp, LSU = 1435–2397 bp, *RPB2* = 485–1080 bp and *TUB2* = 1085–1430 bp) after alignment. *Pleiochaetasetosa* CBS 118.25 / CBS 496.63 was used as the outgroup. Of the 12 new isolates, two isolates clustered with *Stagonosporopsiscaricae* (99% in ML and 1 in PP), three clustered with *Remotididymellaanemophila* (100% in ML and 1 in PP), two clustered with *Paraboeremialitseae* (94% in ML and 1 in PP) and one clustered with *Neoascochytamortariensis* (100% in ML and 1 in PP). One isolate formed a new clade named *N.yunnanensis* and showed a close phylogenetic affinity to *N.rosicola* (MFLUCC 15-0048) in the combined phylogeny and this relationship retrieved 99% ML and 1 PP support. Two isolates formed a new monophyletic clade named *N.zhejiangensis* with high support (100% in ML and 1 in PP). Unfortunately, the non-viability of YCW1124 resulted in the failure of identification, so it was tentatively determined as unidentified species *Neoascochyta* sp. (Fig. 4).

### ﻿Morphology and taxonomy

Based on the multi-locus phylogenetic analysis, six species are delineated as new and their morphological characteristics are described below. In addition, 15 new record species and three known species are noted.

#### ﻿ Didymella coffeae-arabicae

Taxon classificationFungiPleosporalesDidymellaceae

(M. M. Aveskamp et al.) Q. Chen et al., Studies in Mycology. 82: 175. 2015a

B08A81FD-A0F2-54F6-B323-2FE09E787035

##### Description.

see Aveskamp et al. (2009).

##### Materials examined.

China, Yunnan Province, Puer City, Jingdong Yizu Autonomous County, from healthy leaves of *C.sinensis* cv. *Longjing43*, 13 Jun 2020, Y. C. Wang, culture YCW1972.

##### Notes.

*Didymellacoffeae-arabicae* was introduced as *Phomacoffeae-arabicae* before the comprehensive revision of Didymellaceae (Chen et al. 2015a). The sexual morph of *D.coffeae-arabicae* was reported by Samaradiwakara et al. (2023). It forms pseudo-sclerotioid chlamydospores and is easily recognised by its conspicuously wide ostiole and is phylogenetically related to a group that mainly comprises *Peyronellaea* species forming alternarioid-botryoid chlamydospores (Aveskamp et al. 2009). *Didymellacoffeae-arabicae* caused leaf cankers of *Castaneamollissima* in China (Jiang et al. 2021). In the present study, one isolate from healthy tea plant leaves grouped with *D.coffeae-arabicae* with high statistical support (Fig. 2). This is the first report of *D.coffeae-arabicae* isolated from *C.sinensis*.

#### ﻿ Didymella pomorum

Taxon classificationFungiPleosporalesDidymellaceae

(Thüm.) Q. Chen & L. Cai, Studies in Mycology. 82: 179. 2015a

0AABC490-AB35-5F40-B96E-70AC9FE7A64B

##### Description.

see Boerema (1993).

##### Materials examined.

China, Yunnan Province, from diseased leaves of *C.sinensis* cv. *Dalicha*, 22 Jun 2019, Y. C. Wang, culture YCW196.

##### Notes.

*Didymellapomorum* was introduced as *Phomapomorum* before the comprehensive revision of Didymellaceae (Chen et al. 2015a). Chen et al. (2015a) regarded four taxa of the respective *Phomapomorum* varieties, viz. vars. *circinata* (CBS 285.76), *cyanea* (CBS 388.80) and *pomorum* (CBS 539.66) and the species *Ph.triticina* (CBS 354.52) to be conspecific and treated them as a single species *D.pomorum*. Pycnidia produced by this species are usually subglobose-ampulliform with a distinct ostiole (Boerema 1993). It can cause leaf spots on many plants (Boerema 1993; Romero et al. 2021). In the present study, one isolate from diseased tea plant leaves is closely related to *D.sinensis* with high statistical support (Fig. 2). This is the first report of *D.pomorum* isolated from *C.sinensis*.

#### ﻿ Didymella segeticola

Taxon classificationFungiPleosporalesDidymellaceae

(Q. Chen) Q. Chen et al., Studies in Mycology. 87: 138. 2017

C7009A1B-7F69-56DD-A741-CAEECCC6C60C

##### Description.

see Chen et al. (2015b).

##### Materials examined.

China, Jiangsu Province, Yixing City, Zhangzhu Town, Furong Village, from diseased leaves of *C.sinensis* cv. *Longjing43*, 19 Jun 2019, Y. C. Wang, culture YCW109. Zhejiang Province, Lishui City, from diseased leaves of *C.sinensis* cv. *Baiye1*, 22 Jun 2019, Y. C. Wang, culture YCW192. Zhejiang Province, Hangzhou City, from diseased leaves of *C.sinensis* cv. *Longjing43*, 6 Jun 2018, Y. C. Wang, culture YCW1289.

##### Notes.

*Didymellasegeticola* was introduced as *Phomasegeticola* before the comprehensive revision of Didymellaceae (Chen et al. 2015a). Under the current circumstance of Didymellaceae, it belongs to *Didymella*. *Didymellasegeticola* can develop abundant aerial mycelium and black pycnidia on oatmeal agar (OA) plates (Chen et al. 2015b). Zhao et al. (2018) first reported that *D.segeticola* can cause tea leaf spot in the tea plantations in Guizhou Province, which results in leaf fall and a huge loss of tea leaves. In the present study, 171 isolates from diseased tea plant leaves formed a monophyletic subclade, closely related to *D.bellidis* with high statistical support (Fig. 2).

#### ﻿ Didymella sinensis

Taxon classificationFungiPleosporalesDidymellaceae

(Q. Chen) Q. Chen et al., Studies in Mycology. 87: 138. 2017

F204DF33-DE63-569C-B292-304E19884F3C

##### Description.

see Chen et al. (2017).

##### Materials examined.

China, Yunnan Province, Puer City, Jingdong Yizu Autonomous County, from healthy leaves of *C.sinensis*, 13 Jun 2020, Y. C. Wang, culture YCW2118.

##### Notes.

*Didymellasinensis* is closely related to *D.pomorum*. It can be observed from different host plants in a wide range, such as *Cerasuspseudocerasus* (Rosaceae), *Dendrobiumofficinale* (Orchidaceae) and Urticaceae. The sexual morph was characterised by ascomata aggregated, globose to irregular, brown, small and papillate. Asci were bitunicate, clavate to short cylindrical; Ascospores were biseriate, ellipsoidal, straight to slightly curved, hyaline, apex obtuse, medianly 1-septate (Chen et al. 2017). In the present study, eight isolates from healthy tea plant leaves phylogenetically grouped with *D.sinensis* with high statistical support (Fig. 2). This is the first report of *D.sinensis* isolated from *C.sinensis*.

#### ﻿ Didymella yunnanensis

Taxon classificationFungiPleosporalesDidymellaceae

Y. Wang, Y. Tu, X. Chen, H. Jiang, H. Ren, Q. Lu, C. Wei & W. Lv sp. nov.

F8567622-B922-525A-9740-472B505970CD

848984

Fig. 5

##### Etymology.

Named after the location where it was collected, Yunnan Province.

##### Description.

***Sexual morph***: undetermined. ***Asexual morph***: Pycnidia smooth, subglobose to ellipsoidal, hyaline. Conidia ellipsoidal to subcylindrical, pale, smooth- and thin-walled, abundant, generated from pycnidia, aseptate, 4–6.5 × 1.8–2.6 µm (av. = 5.2 ± 0.5 × 2.3 ± 0.2 µm, n = 30). Mycelia sparsely branched from subapical hyphal compartments (lateral branching), septate, hyaline.

##### Culture characteristics.

Colonies on PDA have scarce aerial mycelium reaching 24–27 mm diam. after being cultured for 7 days at 28 °C in the dark, margin regular, olive in the centre, white edges; black on the reverse, white edges. Pycnidia and conidia produced on the colony surface after being cultured for 14 days at 28 °C in the dark. Colonies on OA reaching 18–21 mm diam. after 7 days at 28 °C in the dark, margin regular, aerial mycelium flat, black in the centre, white edges; olive on the reverse, white edges.

**Figure 5.**
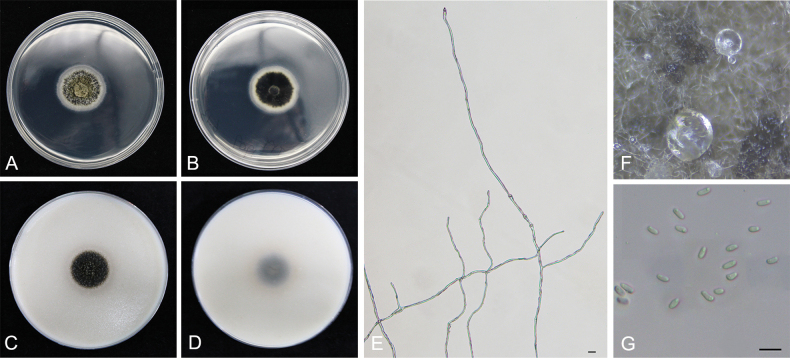
*Didymellayunnanensis*CGMCC 3.24241 (YCW1909) **A, B** colony on PDA (front and reverse) **C, D** colony on OA (front and reverse) **E** myceli **F** pycnidia forming on PDA **G** conidia. Scale bars: 10 μm (**E–G**).

##### Materials examined.

China, Yunnan Province, Puer City, Jingdong Yizu Autonomous County, from healthy leaves of *C.sinensis* cv. *Longjing43*, 16 Jun 2020, Y. C. Wang, Holotype HMAS 352387, culture ex-type CGMCC 3.24241 = YCW1909.

##### Notes.

*Didymellayunnanensis* is closely related to *D.prosopidis* with high statistical support (88%/0.92, ML/PP, Fig. 2). *Didymellayunnanensis* has 85 bp differences in LSU locus from *D.prosopidis*. In addition, *D.yunnanensis* can be distinguished from *D.prosopidis* by the morphological features of conidia and the conidia size of *D.yunnanensis* (4–6.5 × 1.8–2.6 µm) is smaller than that of *D.prosopidis* (5–7 × 2.5–3.5 µm). In the present study, *Didymellayunnanensis* was isolated from healthy tea plant leaves.

#### ﻿ Epicoccum anhuiense

Taxon classificationFungiPleosporalesDidymellaceae

Y. Wang, Y. Tu, X. Chen, H. Jiang, H. Ren, Q. Lu, C. Wei & W. Lv sp. nov.

C70B60BD-2EE5-5D39-BC39-EAD224287330

848998

Fig. 6

##### Etymology.

Named after the location where it was collected, Anhui Province.

##### Description.

***Sexual morph***: undetermined. ***Asexual morph***: Pycnidia smooth, subglobose to ellipsoidal, pale brown, attached to mycelium. Conidia ellipsoidal to subcylindrical, pale yellow to green, smooth- and thin-walled, abundant, generated from pycnidia, composed of a single cell, 10.5–16 × 4.5–8 µm (av. = 13.4 ± 1.4 × 6.3 ± 0.7 µm, n = 30). Mycelia lateral branching, septate, hyaline.

##### Culture characteristics.

Colonies on PDA reaching 75–79 mm diam. after 7 days at 28 °C in the dark, margin regular, covered by floccose aerial mycelium, greyish; reverse pale brown to pale buff, white edges. Pycnidia and conidia produced on the colony surface after being cultured for 14 days at 28 °C in the dark. Colonies on OA reaching 81–85 mm diam. after 7 days at 28 °C in the dark, margin irregular, aerial mycelium flat, whitish; reverse concolorous.

**Figure 6.**
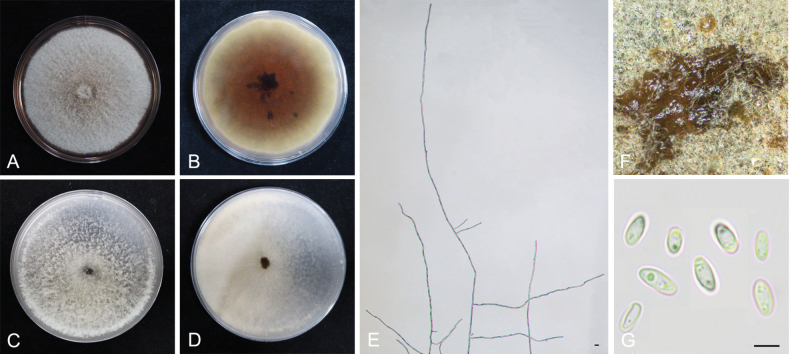
*Epicoccumanhuiense* YCW961 **A, B** colony on PDA (front and reverse) **C, D** colony on OA (front and reverse) **E** mycelia **F** pycnidia forming on PDA **G** conidia. Scale bars: 10 μm (**E–G**).

##### Materials examined.

China, Anhui Province, Anqing City, from diseased leaves of *C.sinensis* cv. *Longjingchangye*, 16 Nov 2019, Y. C. Wang, Holotype HMAS 352388, culture ex-type CGMCC 3.24242 = YCW961. Yunnan Province, Puer City, Jingdong Yizu Autonomous County, from health leaves of *C.sinensis*, 13 Jun 2020, Y. C. Wang, culture ex-type CGMCC 3.24246 = YCW1829.

##### Notes.

*Epicoccumanhuiense* is closely related to *E.latusicollum* with high statistical support (Fig. 3). *Epicoccumanhuiense* has 5 bp differences in the *TUB2* sequence from *E.latusicollum*. In addition, *E.anhuiense* can be distinguished from *E.latusicollum* by the morphological features of its conidia and the conidia size of *E.anhuiense* (10.5–16 × 4.5–8 µm) is larger than that of *D.prosopidis* (4–6.5 × 2–3 µm). In the present study, eight strains were isolated from healthy or diseased tea plant leaves.

#### ﻿ Epicoccum catenisporum

Taxon classificationFungiPleosporalesDidymellaceae

N. Valenzuela-Lopez et al., Studies in Mycology. 90: 30. 2018

98A16F74-3F4E-5702-9233-302B76728C5B

##### Description.

see Valenzuela-Lopez et al. (2018).

##### Materials examined.

China, Jiangxi Province, Nanchang City, from diseased leaves of *C.sinensis* cv. *Zhenong139*, 22 Jun 2019, Y. C. Wang, culture YCW142.

##### Notes.

*Epicoccumcatenisporum* was introduced as *Phomacatenisporum* before the comprehensive revision of *Epicoccum* (Chen et al. 2015a). It was first isolated from a leaf spot of *Oryzasativa* in Guinea-Bissau and morphologically characterised by the production of pycnidia as observed in several other members of *Epicoccum* (Valenzuela-Lopez et al. 2018). Conidiogenous cells were phialidic, hyaline, doliiform or ampulliform and conidia were aseptate, hyaline ovoid or ellipsoidal and guttulate (Valenzuela-Lopez et al. 2018). In the present study, one isolate from diseased tea plant leaves grouped with *E.catenisporum* (CBS 181.80) with high statistical support (Fig. 3). This is the first report of *E.catenisporum* isolated from *C.sinensis*.

#### ﻿ Epicoccum dendrobii

Taxon classificationFungiPleosporalesDidymellaceae

Q. Chen et al., Studies in Mycology. 87: 140. 2017

1AC4C44E-4985-5605-A68C-3A47A3AEF47B

##### Description.

see Chen et al. (2017).

##### Materials examined.

China, Yunnan Province, Puer City, Jingdong Yizu Autonomous County, from healthy leaves of *C.sinensis*, 13 Jun 2020, Y. C. Wang, culture YCW1866.

##### Notes.

*Epicoccumdendrobii* formed a distinct clade, closely related to *E.jingdongense* and *E.puerense* (Fig. 3). It produced typical epicoccoid conidia (multicellular-phragmosporous, verrucose). In the present study, three strains were isolated from healthy or diseased tea plant leaves. This is the first report of *E.dendrobii* isolated from *C.sinensis*.

#### ﻿ Epicoccum draconis

Taxon classificationFungiPleosporalesDidymellaceae

(Berk. ex Cooke) Q. Chen et al., Studies in Mycology. 82: 172. 2015b

6BA65CBF-6626-53E7-B1FD-BCE0971721B8

##### Description.

see de Gruyter et al. (1998).

##### Materials examined.

China, Jiangsu Province, Yixing City, Zhangzhu Town, Furong Village, from diseased leaves of *C.sinensis* cv. *Longjing43*, 19 Jun 2019, Y. C. Wang, culture YCW101.

##### Notes.

*Epicoccumdraconis* was introduced as *Phyllostictadraconis* and *Phomadraconis* previously (Chen et al. 2017). It formed a new combination by the ellipsoidal conidia (Chen et al. 2017). In the present study, two isolates from diseased tea plant leaves grouped with *E.draconis* with high statistical support (Fig. 3). This is the first report of *E.draconis* causing leaf blight on *C.sinensis*.

#### ﻿ Epicoccum italicum

Taxon classificationFungiPleosporalesDidymellaceae

Q. Chen et al., Studies in Mycology. 87: 144. 2017

9C2BF591-6E2B-591B-8779-39EB526EAF58

##### Description.

see Chen et al. (2017).

##### Materials examined.

China, Yunnan Province, Puer City, Jingdong Yizu Autonomous County, from healthy leaves of *C.sinensis*, 13 Jun 2020, Y. C. Wang, culture YCW2005.

##### Notes.

Phylogenetically, *Epicoccumitalicum* formed a distinct lineage closely related to *E.oryzae* (Fig. 3). *Epicoccumitalicum* produced epicoccoid conidia and clavate conidiomata (Chen et al. 2017). It was first isolated from seedlings of *Accasellowiana* in Italy (Chen et al. 2017) and reported in the dairy setting (Rodríguez et al. 2023). In addition, this species significantly reduced both leaf area of soybean consumed aboveground by caterpillars and number of cysts produced belowground by nematodes (Rivera-Vega et al. 2022). In the present study, one strain was isolated from healthy tea plant leaves. This is the first report of *E.italicum* isolated from *C.sinensis*.

#### ﻿ Epicoccum jingdongense

Taxon classificationFungiPleosporalesDidymellaceae

Y. Wang, Y. Tu, X. Chen, H. Jiang, H. Ren, Q. Lu, C. Wei & W. Lv sp. nov.

46008A6A-EE2C-5CE0-9A9E-3CFD9B9D3C3E

849000

Fig. 7

##### Etymology.

Named after the location where it was collected, Jingdong Yizu Autonomous County.

##### Description.

***Sexual morph***: undetermined. ***Asexual morph***: Pycnidia smooth, subglobose, pale brown. Conidia ellipsoidal to subcylindrical, pale yellow, smooth, generated from pycnidia, aseptate, 7.1–16 × 4–9 µm (av. = 10.7 ± 1.2 × 5.4 ± 0.6 µm, n = 30). Mycelia extensively branched from subapical hyphal compartments, septate, hyaline.

##### Culture characteristics.

Colonies on PDA reaching 35–42 mm diam. after 7 days at 28 °C in the dark, margin irregular, aerial mycelium flat, pale brown to rosy, white edges; reverse black to brown, pale buff edges. Pycnidia and conidia produced on the colony surface after being cultured for 14 days at 28 °C in the dark. Colonies on OA reaching 49–55 mm diam. after 7 days at 28 °C in the dark, margin regular, aerial mycelium flat, pale buff to whitish; reverse concolorous.

**Figure 7.**
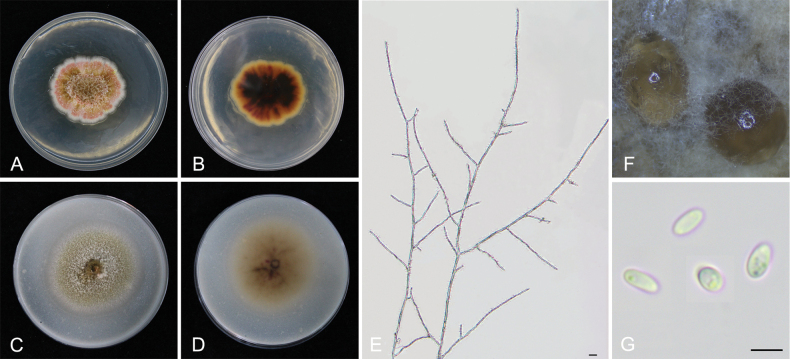
*Epicoccumjingdongense* YCW1868 **A, B** colony on PDA (front and reverse) **C, D** colony on OA (front and reverse) **E** mycelia **F** pycnidia forming on PDA **G** conidia. Scale bars: 10 μm (**E–G**).

##### Materials examined.

China, Yunnan Province, Puer City, Jingdong Yizu Autonomous County, from healthy leaves of *C.sinensis*, 13 Jun 2020, Y. C. Wang, Holotype HMAS 352389, culture ex-type CGMCC 3.24247 = YCW1868. Yunnan Province, Puer City, Jingdong Yizu Autonomous County, from healthy leaves of *C.sinensis*, 13 Jun 2020, Y. C. Wang, culture ex-type CGMCC 3.24248 = YCW1937.

##### Notes.

*Epicoccumjingdongense* is closely related to *E.dendrobii* and *E.puerense* with high statistical support (83%/0.99, ML/PP, Fig. 3). *Epicoccumpuerense* differs in 1 bp in ITS and 40 bp in *TUB2* from *E.dendrobii*. The conidia size is larger than that of *E.dendrobii*. In the present study, six strains were isolated from healthy tea plant leaves. It was isolated and identified from tea plant for the first time.

#### ﻿ Epicoccum latusicollum

Taxon classificationFungiPleosporalesDidymellaceae

Q. Chen et al., Studies in Mycology. 87: 144. 2017

762FE0EA-0ABC-5FE0-9552-F80E89B5E5E3

##### Description.

see Chen et al. (2017).

##### Materials examined.

China, Yunnan Province, Puer City, Jingdong Yizu Autonomous County, from healthy leaves of *C.sinensis*, 13 Jun 2020, Y. C. Wang, culture YCW1921.

##### Notes.

Isolates of *Epicoccumlatusicollum* were clustered into a sister clade to *E.poaceicola* and *E.sorghi* (Fig. 3). Pycnidia were black-brown and mostly spheroid and conidia were ellipsoidal to oblong, aseptate and hyaline (Chen et al. 2017; Li et al. 2023). It was first discovered from *Acerpalmatum* (Aceraceae), *Camelliasinensis* (Theaceae), *Podocarpusmacrophyllus* (Podocarpaceae) and *Vitexnegundo* (Verbenaceae) (Chen et al. 2017). As a phytopathogen, it can cause leaf spot, leaf blight and stalk rot on many plants (Xu et al. 2022; Li et al. 2023; Wang et al. 2023). In the present study, three strains were isolated from healthy tea plant leaves.

#### ﻿ Epicoccum mackenziei

Taxon classificationFungiPleosporalesDidymellaceae

S. C. Jayasiri et al., Mycosphere 8: 1093. 2017

FFC972D1-1AAC-5534-9BB8-7ED268998484

##### Description.

see Jayasiri et al. (2017).

##### Materials examined.

China, Yunnan Province, Puer City, Jingdong Yizu Autonomous County, from healthy leaves of *C.sinensis*, 13 Jun 2020, Y. C. Wang, culture ex-type CGMCC 3.24244 = YCW1965 and culture ex-type CGMCC 3.24245 = YCW1967.

##### Notes.

*Epicoccummackenziei* formed a distinct clade basal to *E.endophyticum* (Fig. 3). It was found as the sexual morph in nature and as chlamydospores in culture. Zhang et al. (2023) first reported that *E.mackenziei* caused dark brown spot of tea leaf in China. In the present study, two strains were isolated from healthy tea plant leaves.

#### ﻿ Epicoccum oryzae

Taxon classificationFungiPleosporalesDidymellaceae

S. Ito & Iwadare, Report of the Hokkaido Prefectural Agricultural Experiment Station 31: 1. 1934

CC5BE773-9ECB-542E-A08D-34696B7F3209

##### Description.

see Hou et al. (2020b).

##### Materials examined.

China, Yunnan Province, Puer City, Jingdong Yizu Autonomous County, from healthy leaves of *C.sinensis*, 13 Jun 2020, Y. C. Wang, culture YCW2010.

##### Notes.

*Epicoccumoryzae* was synonymised as *E.nigrum* previously (Schol – Schwarz 1959). It was resurrected as a separate species, distant from *E.nigrum* and CBS 173.34 was proposed as the ex-neotype of *E.oryzae* (Hou et al. 2020b). *Epicoccumoryzae* is characterised by “olivaceous hyphae, globose or subglobose sporodochia and globose, subglobose or pyriform, granular, verrucose, olivaceous conidia, consisting of one to five cells” (Hou et al. 2020b). It clustered into a sister clade to *E.endophyticum* and *E.mackenziei* (Fig. 3). In the present study, one isolate from healthy tea plant leaves grouped with *E.draconis* (CBS 173.34 and CBS 174.34) with high statistical support (Fig. 3). This is the first report of *E.oryzae* isolated from *C.sinensis*.

#### ﻿ Epicoccum poaceicola

Taxon classificationFungiPleosporalesDidymellaceae

Thambugala & K.D. Hyde, Mycosphere. 8: 711. 2017

E7D57484-0D9B-5E90-8B10-CFF791434C4F

##### Description.

see Thambugala et al. (2017).

##### Materials examined.

China, Yunnan Province, Puer City, Jingdong Yizu Autonomous County, from healthy leaves of *C.sinensis*, 13 Jun 2020, Y. C. Wang, culture YCW1948.

##### Notes.

*Epicoccumpoaceicola* is described as a new phoma-like species, based on phylogenetic analysis. It formed a distinct lineage closely related to *E.sorghi* (Fig. 3). Conidia produced by *E.poaceicola* were ellipsoidal to cylindrical and sometimes with small guttules (Thambugala et al. 2017). *Epicoccumpoaceicola* can cause leaf spot in bamboo, camphor tree and eggplant (Liu et al. 2020; Li et al. 2022; Aementado and Balendres 2023). In the present study, seven strains were isolated from healthy tea plant leaves. This is the first report of *E.poaceicola* isolated from *C.sinensis*.

#### ﻿ Epicoccum puerense

Taxon classificationFungiPleosporalesDidymellaceae

Y. Wang, Y. Tu, X. Chen, H. Jiang, H. Ren, Q. Lu, C. Wei & W. Lv sp. nov.

049807CF-8E14-59BC-A2B6-3DE0622DDA56

848999

Fig. 8

##### Etymology.

Named after the location where it was collected, Puer City.

##### Description.

***Sexual morph***: undetermined. ***Asexual morph***: Pycnidia smooth, subglobose to ellipsoidal, hyaline. Conidia were not of uniform size, ellipsoidal to subcylindrical, pale yellow to green, smooth- and thin-walled, abundant, generated from pycnidia, aseptate, 6.8–15 × 3.6–7.2 µm (av. = 9.7 ± 1.9 × 4.7 ± 0.7 µm, n = 30). Mycelia lateral branching, septate, hyaline.

##### Culture characteristics.

Colonies on PDA reaching 32–41 mm diam. after 7 days at 28 °C in the dark, margin irregular, aerial mycelium flat, olivaceous to buff, white edges; reverse black to brown, pale buff edges. Pycnidia and conidia produced on the colony surface after cultured for 14 days at 28 °C in the dark. Colonies on OA reaching 51–58 mm diam. after 7 days at 28 °C in the dark, margin regular, aerial mycelium flat, rosy to pale green, white edges; reverse pale buff to whitish.

**Figure 8.**
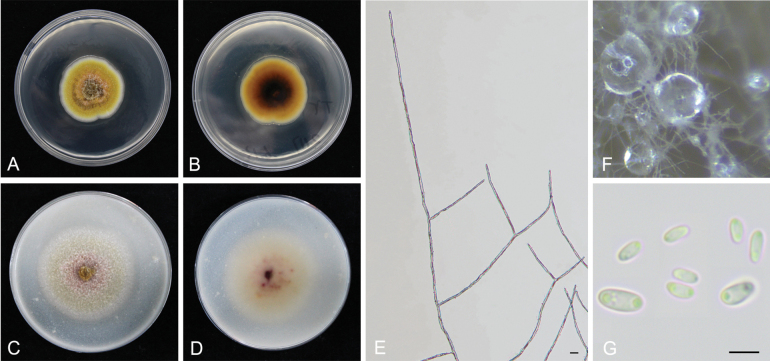
*Epicoccumpuerense* YCW2117 **A, B** colony on PDA (front and reverse) **C, D** colony on OA (front and reverse) **E** mycelia **F** pycnidia forming on PDA **G** conidia. Scale bars: 10 μm (**E–G**).

##### Materials examined.

China, Yunnan Province, Puer City, Jingdong Yizu Autonomous County, from diseased leaves of *C.sinensis*, 13 Jun 2020, Y. C. Wang, Holotype HMAS 352390, culture ex-type CGMCC 3.24249 = YCW2117. Yunnan Province, from healthy leaves of *C.sinensis* cv. *Dalicha*, 22 Jun 2019, Y. C. Wang, culture ex-type CGMCC 3.24243 = YCW224.

##### Notes.

*Epicoccumpuerense* is closely related to *E.dendrobii* with high statistical support (Fig. 3). *Epicoccumpuerense* has 1 bp difference in ITS from *E.dendrobii*. The conidia size is larger than that of *E.dendrobii*. In the present study, five strains were isolated from healthy or diseased tea plant leaves. It was isolated and identified from tea plant for the first time.

#### ﻿ Epicoccum rosae

Taxon classificationFungiPleosporalesDidymellaceae

D. N. Wanasinghe et al., Fungal Diversity. 89: 29. 2018

A1AA1902-3985-5007-9284-45EE965626CD

##### Description.

see Wanasinghe et al. (2018b).

##### Materials examined.

China, Hubei Province, Wuhan City, Jiangxia District, from diseased leaves of *C.sinensis* cv. *Yulv*, 10 Jul 2019, Y. C. Wang, culture YCW331.

##### Notes.

*Epicoccumrosae* had pycnidial conidiomata with hyaline conidia and hyphomycetous dark sporodochia with branched conidiophores and verruculose, muriform chlamydospores. It formed a distinct lineage closely related to *E.tobaicum* (Fig. 3). In the present study, three strains were isolated from diseased tea plant leaves. This is the first report of *E.rosae* isolated from *C.sinensis*.

#### ﻿ Epicoccum tobaicum

Taxon classificationFungiPleosporalesDidymellaceae

(Svilv.) L.W. Hou et al., Studies in Mycology. 96: 348. 2020

EF72C4FE-53D9-5CEC-84FA-D5A91FEBD2E7

##### Description.

see von Szilvinyi (1936).

##### Materials examined.

China, Anhui Province, Huangshan City, from diseased leaves of *C.sinensis* cv. *Zhonghuang1*, 2 Jul 2019, Y.C. Wang, culture YCW372.

##### Notes.

*Epicoccumtobaicum* was synonymised as *E.nigrum* previously (Hou et al. 2020b). It was resurrected as a separate species, distant from *E.nigrum* (Hou et al. 2020b). Conidia were globular to pear-shaped, dark, verrucose and multicellular (Han et al. 2021). It formed a distinct lineage closely related to *E.rosae* (Fig. 3). This species as a pathogen was isolated from diseased leaves showing leaf spot of flowering cherry and oat (Han et al. 2021; Jeong et al. 2022a). In the present study, five strains were isolated from diseased tea plant leaves. This is the first report of *E.tobaicum* isolated from *C.sinensis*.

#### ﻿ Neoascochyta mortariensis

Taxon classificationFungiPleosporalesDidymellaceae

L.W. Hou et al., Studies in Mycology. 96: 391. 2020

88B3E7EB-DC6D-56B5-8425-1C1A033D584E

##### Description.

see Hou et al. (2020b).

##### Materials examined.

China, Zhejiang Province, Hangzhou City, from healthy leaves of *C.sinensis* cv. *Longjing43*, 16 Nov. 2017 Y. C. Wang, culture ex-type CGMCC 3.24251 = YCW1346.

##### Notes.

*Neoascochytamortariensis* was introduced as *Didymellagraminicola* previously. It was described as a new species in *Neoascochyta*, distant from the authentic culture of *D.graminicola* (currently: *Neoascochytagraminicola*) (Hou et al. 2020b). *Neoascochytamortariensis* was first isolated from *Oryzasativa* in Italy and formed colonies on PDA covered by dense felty aerial mycelium (Hou et al. 2020b). It formed a distinct lineage closely related to *N.tardicrescens* (Fig. 4). In the present study, one strain was isolated from diseased tea plant leaves. This is the first report of *N.mortariensis* isolated from *C.sinensis*.

#### ﻿ Neoascochyta yunnanensis

Taxon classificationFungiPleosporalesDidymellaceae

Y. Wang, Y. Tu, X. Chen, H. Jiang, H. Ren, Q. Lu, C. Wei & W. Lv sp. nov.

72A64BE6-0780-5DAB-9871-522A4C052DDB

849001

Fig. 9

##### Etymology.

Named after the location where it was collected, Yunnan Province.

##### Description.

***Sexual morph***: undetermined. ***Asexual morph***: Pycnidia smooth, subglobose to ellipsoidal, hyaline. Conidia ellipsoidal to subcylindrical, pale yellow to green, smooth- and thin-walled, abundant, generated from pycnidia, aseptate, 8.5–11.7 × 4.5–7 µm (av. = 9.9 ± 0.9 × 5.4 ± 0.6 µm, n = 30). Mycelia lateral branching, septate, hyaline.

##### Culture characteristics.

Colonies on PDA reaching 42–45 mm diam. after 7 days 28 °C in the dark, margin regular, aerial mycelium flat, whitish; reverse black to pale buff. Pycnidia and conidia produced on the colony surface after being cultured for 14 days at 28 °C in the dark. Colonies on OA reaching 34 – 39 mm diam. after 7 days at 28 °C in the dark, margin irregular, aerial mycelium flat, black in the centre, white edges; reverse concolorous.

**Figure 9.**
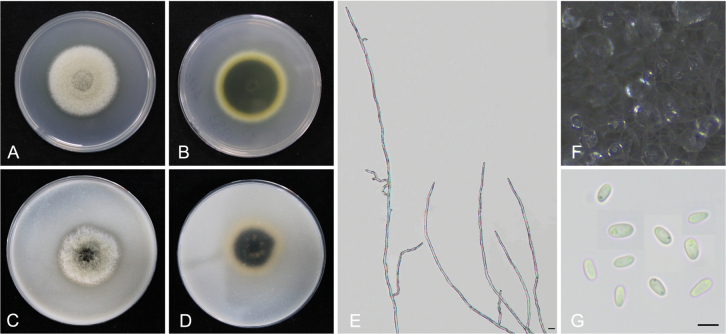
*Neoascochytayunnanensis* YCW1883 **A, B** colony on PDA (front and reverse) **C, D** colony on OA (front and reverse) **E** mycelia **F** pycnidia forming on PDA **G** conidia. Scale bars: 10 μm (**E–G**).

##### Materials examined.

China, Yunnan Province, Puer City, Jingdong Yizu Autonomous County, from healthy leaves of *C.sinensis*, 13 Jun 2020, Y. C. Wang, Holotype HMAS 352391, culture ex-type CGMCC 3.24253 = YCW1883.

##### Notes.

*Neoascochytayunnanensis* is closely related to *N.rosicola* with high statistical support (99%/1, ML/PP, Fig. 4). *Neoascochytayunnanensis* has 2 bp differences in ITS from *N.rosicola*. In the present study, one strain was isolated from healthy tea plant leaves. It was isolated and identified from tea plant for the first time.

#### ﻿ Neoascochyta zhejiangensis

Taxon classificationFungiPleosporalesDidymellaceae

Y. Wang, Y. Tu, X. Chen, H. Jiang, H. Ren, Q. Lu, C. Wei & W. Lv sp. nov.

BD289EE0-6066-5714-BF22-7C7958687CC4

849002

Fig. 10

##### Etymology.

Named after the location where it was collected, Zhejiang Province.

##### Description.

***Sexual morph***: undetermined. ***Asexual morph***: Pycnidia smooth, subglobose to ellipsoidal, hyaline. Conidia biconical to subcylindrical, hyaline, smooth- and thin-walled, abundant, generated from pycnidia, aseptate, 4.8–6.5 × 2.9–4.2 µm (av. = 5.6 ± 0.5 × 3.6 ± 0.3 µm, n = 30). Mycelia lateral branching or uniaxial branching, septate, hyaline.

**Figure 10.**
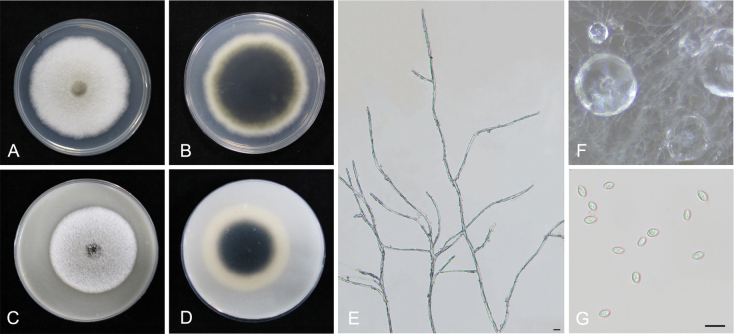
*Neoascochytazhejiangensis* YCW1107 **A, B** colony on PDA (front and reverse) **C, D** colony on OA (front and reverse) **E** mycelia **F** pycnidia forming on PDA **G** conidia. Scale bars: 10 μm (**E–G**).

##### Culture characteristics.

Colonies on PDA reaching 65–69 mm diam. after 7 days at 28 °C in the dark, margin regular, aerial mycelium flat, whitish; reverse black, white edges. Pycnidia and conidia produced on the colony surface after being cultured for 14 days at 28 °C in the dark. Colonies on OA reaching 53–57 mm diam. after 7 days at 28 °C in the dark, margin regular, aerial mycelium flat, whitish; reverse olivaceous, white edges.

##### Materials examined.

China, Zhejiang Province, Hangzhou City, from diseased leaves of *C.sinensis* cv. *Longjing43*, Jun 2014, Y. C. Wang, Holotype HMAS 352392, culture ex-type CGMCC 3.24250 = YCW1107. Yunnan Province, from diseased leaves of *C.sinensis*, 23 Mar 2020, Y. C. Wang, culture CGMCC 3. YCW1361.

##### Notes.

*Neoascochytazhejiangensis* is closely related to *N.cylindrispora* with high statistical support (82%/77, ML/PP, Fig. 4). *Neoascochyta Cylindrispora* differs in 1 bp in ITS, 16 bp in *TUB2* and 95 bp in LSU from *N.zhejiangensis*. In the present study, two strains were isolated from healthy tea plant leaves.

#### ﻿ Paraboeremia litseae

Taxon classificationFungiPleosporalesDidymellaceae

J. R. Jiang et al., Mycological Progress. 16: 291. 2017

5170F84E-C634-5840-8B05-8971B2744A03

##### Description.

see Jiang et al. (2017).

##### Materials examined.

China, Yunnan Province, from diseased leaves of *C.sinensis*, 23 Mar 2020, Y. C. Wang, culture YCW1356 and culture YCW1363.

##### Notes.

Isolates of *Paraboeremialitseae* clustered into a sister clade to *P.selaginellae* (Fig. 5). It was first isolated from *Litsea* sp. (Jiang et al. 2017). Conidia produced by *P.litseae* are oblong to ellipsoidal and aseptate with two large polar guttules (Jiang et al. 2017). This species as an endophytic fungus in *Coptischinensis* exhibited obvious inhibition against methicillin-resistant *Staphylococcusaureus* (Ming et al. 2022). In the present study, two strains were isolated from diseased tea plant leaves. This is the first report of *P.litseae* causing leaf blight on *C.sinensis*.

#### ﻿ Remotididymella anemophila

Taxon classificationFungiPleosporalesDidymellaceae

A. L. Yang et al., International Journal of Systematic Evolutional Microbiology. 71: 10. 2021

C955BD1F-7216-5620-A81E-92E5F7E9902C

##### Description.

see Yang et al. (2021).

##### Materials examined.

China, Anhui Province, Huangshan City, from diseased leaves of *C.sinensis* cv. *Fenglixiang*, 2 Jul 2019, Y. C. Wang, culture YCW499. Zhejiang Province, Hangzhou City, from diseased leaves of *C.sinensis* cv. *Longjing43*, Jun 2014, Y. C. Wang, culture YCW1118.

##### Notes.

*Remotididymellaanemophila* was clustered into a sister clade to *R.bauhiniae* (Fig. 4), characterised by shorter ascospores, longer asci and larger conidia. It was first isolated from canopy air of *Ageratinaadenophora* (Spreng.) in China (Yang et al. 2021). In the present study, three strains were isolated from diseased tea plant leaves. This is the first report of *R.anemophila* causing leaf blight on *C.sinensis*.

#### ﻿ Stagonosporopsis caricae

Taxon classificationFungiPleosporalesDidymellaceae

(Sydow & P. Sydow) M. M. Aveskamp et al., Studies in Mycology. 65: 45. 2010

A2CEAE8D-F0FD-57F8-A059-B716231D5255

##### Description.

see Sivanesan (1990).

##### Materials examined.

China, Yunnan Province, Puer City, Jingdong Yizu Autonomous County, from healthy leaves of *C.sinensis*, 13 Jun 2020, Y. C. Wang, culture YCW1928. Yunnan Province, Puer City, Jingdong Yizu Autonomous County, from healthy leaves of *C.sinensis*, 13 Jun 2020, Y. C. Wang, culture YCW1977.

##### Notes.

*Stagonosporopsiscaricae* was synonymised as *Phomacaricae* with *Mycosphaerellacaricae* previously (Sivanesan 1990). It formed a distinct lineage in *Stagonosporopsis* (Fig. 4). Zhang et al. (2022) observed its sexual morph and is characterised by ascomata pseudothecioid, subglobose, 121 × 142 μm, ostiolate, walls of brown *textura angularis* and smooth. Asci were bitunicate, cylindrical to clavate, 7 × 90 μm, 8-spored, ascospores elliptical, straight to slightly curved, 5 × 17 μm, 1-septate, constricted at the septum, sub-hyaline and smooth. As one of three *Stagonosporopsis* species, *S.caricae* caused gummy stem blight (Jeong et al. 2022b; Seblani et al. 2023). In the present study, two isolates from healthy tea plant leaves grouped with *S.caricae* with high statistical support (Fig. 4). This is the first report of *S.caricae* isolated from *C.sinensis*.

### ﻿Pathogenicity tests

**Figure 11.**
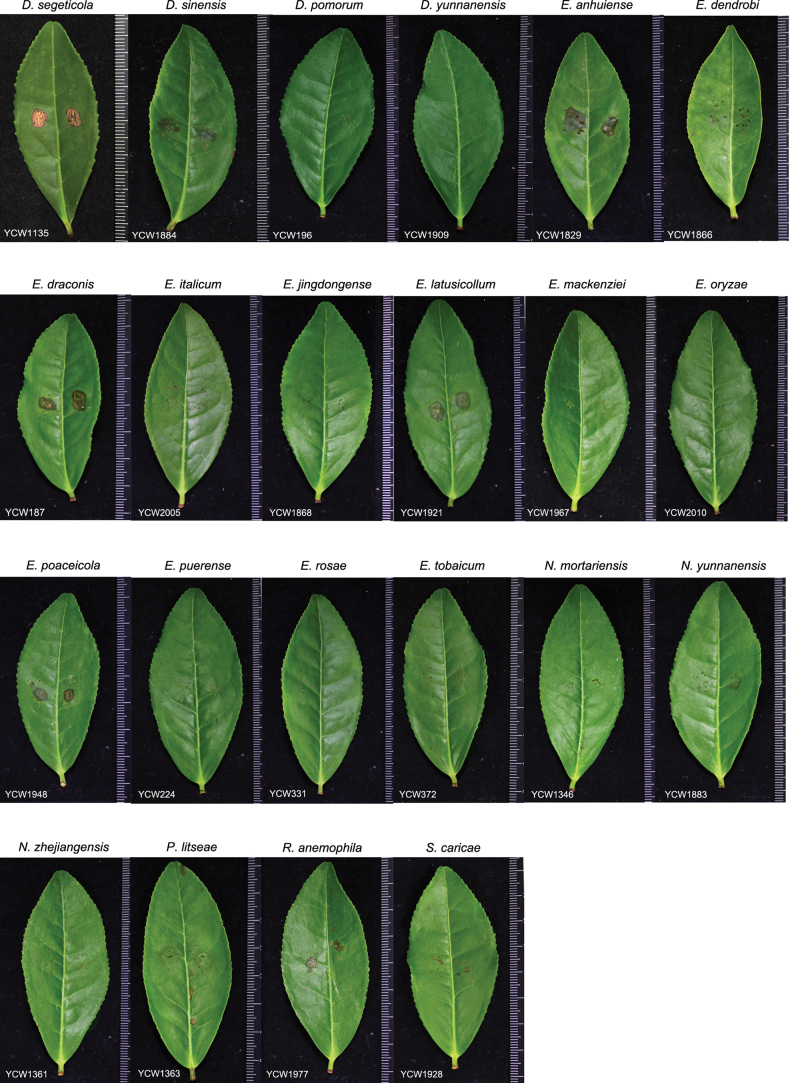
Symptoms of Didymellaceae family strains on tea plant leaves at 3 days after inoculation.

**Figure 12.**
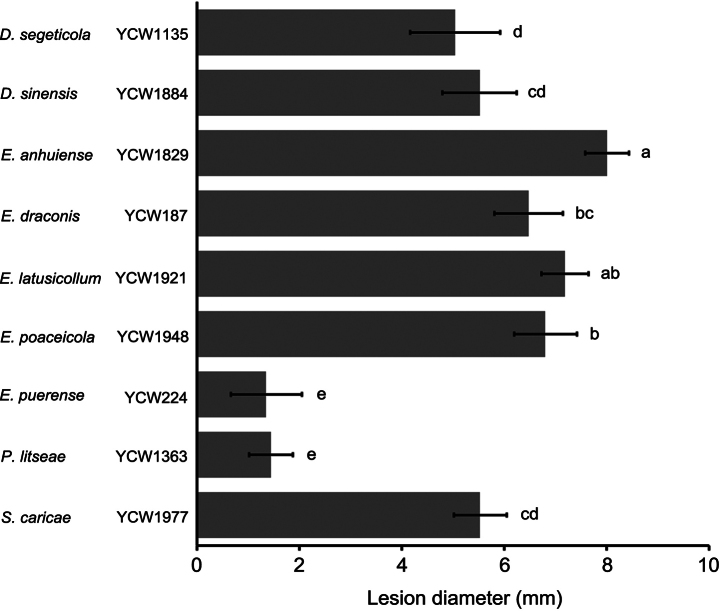
Lesion diameters of Didymellaceae family strains on tea plant leaves at 3 days after inoculation. Error bars represent standard deviation.

To determine the pathogenicity of isolates from these 22 species, 36 representative isolates were selected for the analysis on the healthy leaves of *C.sinensis* cv. *Longjing43* with the wound-inoculation method. Amongst the tested strains, the sizes of necrotic lesions caused by the strain YCW1829 of *E.anhuiense* were largest (av. 8.00 ± 0.42 mm); on the contrary, the size of that caused by the strain YCW224 of *E.puerense* was smallest (av. 1.35 ± 0.70 mm) (Figs 11, 12). *Didymellasegeticola*, *E.draconis*, *E.latusicollum* and *E.poaceicola* could also cause necrotic lesions on the inoculated leaves. Furthermore, the other strains caused no necrotic lesions on tea plant leaves (Fig. 11). The results indicated that *E.anhuiense* had the strongest virulence; on the contrary, *E.puerense* displayed the weakest virulence. In addition, *D.pomorum*, *D.yunnanensis*, *E.dendrobii*, *E.italicum*, *E.mackenziei*, *E.oryzae*, *E.rosae*, *E.tobaicum*, *E.jingdongense*, *N.mortariensis*, *N.yunnanensis*, *N.zhejiangensis* and *R.anemophila* were not pathogenic to tea plants.

### ﻿Geographical distribution

To explore the geographical distribution of Didymellaceae family strains associated with *C.sinensis* in China, we combined our data with these from Chen et al. (2017) and Ren et al. (2019) for the analysis (Table 2). Amongst the 240 isolates that we collected from ten provinces in China, most of the isolates were distributed in Yunnan Province. Amongst the 25 species, *D.segeticola* (171 isolates in this study and 14 isolates from Ren et al. (2019) had the widest geographical distribution, in nine provinces. Fourteen species, *D.coffeae*-*arabicae*, *D.pomorum*, *D.sinensis*, *D.yunnanensis*, *E.dendrobii*, *E.italicum*, *E.jingdongense*, *E.mackenziei*, *E.oryzae*, *E.poaceicola*, *E.puerense*, *N.rosicola*, *P.litseae* and *S.caricae*, were only distributed in Yunnan Province. One species, *E.catenisporum*, was only distributed in Jiangxi Province. These results suggest that *D.segeticola* as the most widely distributed species may be the dominant species causing leaf blight disease in tea plants.

**Table 2.** Geographical distribution of Didymellaceae family associated with *C.sinensis* in China.

Species	Collecting location									
	AH	GD	GZ	HB	HN	JS	JX	SC	YN	ZJ
*D.coffeae-arabicae*									√	
*D.pomorum*									√	
*D.segeticola*	√	√	√⊥	√		√	√	√	√	√
*D.sinensis*									√	
*D.yunnanensis*									√	
*E.anhuiense*	√								√	
*E.catenisporum*							√			
*E.dendrobii*									√	
*E.draconis*						√				√
*E.italicum*									√	
*E.jingdongense*									√	
*E.latusicollum*							*		√	
*E.mackenziei*									√	
*E.oryzae*									√	
*E.poaceicola*									√	
*E.puerense*									√	
*E.rosae*	√			√		√				
*E.sorghinum*							*			√
*E.tobaicum*	√			√	√					√
*N.mortariensis*										√
*N.rosicola*									√	
*N.zhejiangensis*									√	√
*P.litseae*									√	
*R.anemophila*	√									√
*S.caricae*									√	

AH: Anhui; GD: Guangdong; GZ: Guizhou; HB: Hubei; HN: Henan; JS: Jiangsu; JX: Jiangxi; SC: Sichuan; YN: Yunnan; ZJ: Zhejiang. This table includes data from Chen et al. (2017) (*), Ren et al. (2019) (⊥), and our study (√).

## ﻿Discussion

In this study, 240 isolates were obtained from tea plant leaves in ten provinces of four major tea regions (southwest China, south China, south Yangtze and north Yangtze) in China (Yang et al. 2023b). Based on the multi-locus (ITS, LSU, *RPB2* and *TUB2*) sequences, three phylogenetic trees were constructed to identify the species of the tested isolates. Six novel species, named *Didymellayunnanensis*, *Epicoccumanhuiense*, *Epicoccumjingdongense*, *Epicoccumpuerense*, *Neoascochytayunnanensis* and *Neoascochytazhejiangensis*, were identified and their morphological characteristics were described in detail (Figs 4–9). As one of the most species-rich genera in the Didymellaceae, *Didymella* was introduced by Saccardo (1880) with *Didymellaexigua* (Niessl) Sacc. as the type species of the genus (Thambugala et al. 2017; Wang et al. 2021). Most species in the genus produced chlamydospores in culture (Chen et al. 2015a), whereas *D.yunnanensis* as one novel species of *Didymella* did not form chlamydospores on PDA or OA cultures (Fig. 5), which may be the result of suitable culture conditions in the incubator not being favourable for the production of the resting spores. Pycnidia of *D.yunnanensis* formed on PDA was smooth, subglobose to ellipsoidal, hyaline, which conflicts with the pigmented outer wall of pycnidia of *Didymella* genus (Chen et al. 2015a). However, based on multi-locus phylogenetic analyses, *D.yunnanensis* belonged to this genus as a novel species. We believed that multi-locus phylogenetic analyses were the more reliable method to clarify the genetic delimitation in Didymellaceae compared with the morphological observations. Here, we provide phylogenetic trees for *Didymella*, *Epicoccum*, *Neoascochyta*, *Paraboeremia*, *Remotididymella* and *Stagonosporopsis* using as much vouchered sequence data as possible. Six new species and 15 new records are proposed herein with support from our analysis of ITS, LSU, *RPB2* and *TUB2* sequences.

The genus *Epicoccum* is known as a hyphomycetous asexual morph in the Didymellaceae family (Hyde et al. 2013). However, it was emended with coelomycetous synanamorph by Chen et al. (2015a) and five *Phoma* species were recombined into the genus, based on multi-gene phylogenetic analysis (Thambugala et al. 2017). *Epicoccumanhuiense* is phylogenetically distinct from other *Epicoccum* species with close phylogenetic affinity to *E.latusicollum* (5 bp difference within the *TUB2* sequence). *Epicoccumjingdongense* and *E.puerense* are also phylogenetically distinct from other *Epicoccum* species with close phylogenetic affinity to *E.dendrobii* (40 bp difference within the *TUB2* sequence and 1 bp difference within the ITS sequence, respectively). Asexual morphs of the three novel species accommodated in *Epicoccum* were also determined and formed the coelomycetous asexual morphs (Figs 6–8), which is consistent with the characteristics of *Epicoccum* coelomycetous synasexual stage that is characterised by the formation of doliiform to flask-shaped conidiogenous cells that produce unicellular, hyaline conidia under culture conditions (Aveskamp et al. 2010; Jayasiri et al. 2017). Therefore, these species are introduced, based on the synasexual morphs and phylogenetic data.

In *Neoascochyta*, three different groups are observable based on conidial morphology: species with one-septate conidia, such as *N.dactylidis*, *N.europaea*, *N.exitialis* and *N.graminicola*; species with mainly one-septate conidia, but occasionally aseptate, such as *N.argentina*, *N.cylindrispora*, *N.desmazieri*, *N.rosicola*, *N.tardicrescens* and *N.triticicola*; and species with aseptate conidia, such as *N.fuci*, *N.paspali* and *N.soli* (Gonçalves et al. 2020). Two novel species, *N.yunnanensis* and *N.zhejiangensis*, produced aseptate conidia (Figs 9G, 10G), which fit within the last group. All the same, *N.yunnanensis* and *N.zhejiangensis* phylogenetically have a close relationship with *N.rosicola* and *N.cylindrispora*, respectively (Fig. 4). Conidia produced by *N.zhejiangensis* were hyaline, biconical to subcylindrical (Fig. 10G), keeping consistent with the conidial characteristics of *Neoascochyta* genus. By contrast, *N.yunnanensis* formed pale yellow conidia (Fig. 9G), which was a typical characteristic of *Neoascochyta* conidia. Besides, pycnidia of the two species formed on PDA was hyaline (Figs 9F, 10F), which is also a non-representative characteristic. This may be due to the culture conditions. The majority of *Neoascochyta* species was found in association with various Poaceae plant species, appearing to have some host preference (Gonçalves et al. 2020). In this study, we reported two novel species isolated from the tea plant for the first time.

*Didymella* and *Neoascochyta* genera have sexual morphs (Woudenberg et al. 2009; Jayasiri et al. 2017). However, sexual morphs of the isolates belonging to two genera were not observed under culture conditions and then undetermined. In the future, the detailed description of sexual morphs of the isolates, especially the three novel species *D.yunnanensis*, *N.yunnanensis* and *N.zhejiangensis*, will provide more morphological evidence for the identification of the novel species.

Amongst six new species in this study, most isolates were obtained from Yunnan Province (Table 2). Yunnan Province, as the oldest tea region in China, is rich in tea plant resources and is also the centre of fungi biodiversity. Molecular evidence suggested that many fungi belonging to the family Didymellaceae may be seedborne and can co-spread with the host through seeds (Fang et al. 2021; Yang et al. 2023a). Therefore, we speculated that Yunnan as the birthplace of tea plants has more abundant germplasm resources and is prone to fungal transmission. The remaining isolates were collected from Zhejiang and Anhui Provinces (Table 2), which provide the most suitable environment for tea plant growth. This warm and humid climate are also conducive to the rapid growth of fungi (Du et al. 2012).

More than half of the strains isolated from tea plants were clustered into *Didymellasegeticola* species, indicating that this species in Didymellaceae family is probably more dominant in tea plants. They were isolated from diseased tea plant leaves and had strong virulence (Figs 11, 12), suggesting that *D.segeticola* may be the causal agent of foliar diseases in tea plants. *Didymella* species have been reported to cause leaf spot on many plants, such as *Angelicadahurica* (Xu et al. 2016), *Bellisperennis* (Chen et al. 2015a), *Chrysanthemummorifolium* (Liu et al. 2019), *C.sinensis* (Ren et al. 2019; Wang et al. 2021), *Eleocharisdulcis* (Lv et al. 2011), *Lodiummultiflorum* (Liu et al. 2022) and *Zanthoxylumbungeanum* (Yang et al. 2022). Especially, Ren et al. (2019) have also proved that *D.segeticola* is a causal agent of leaf spot on tea plants in China. However, the morphological characteristics of *D.segeticola* shared some similarities with those of *Disculatheae-sinensis*, the causal agent of tea anthracnose (Moriwaki and Sato 2009), especially the conidial morphology. We thus speculated that *D.segeticola* could also be the pathogen causing anthracnose on tea plant leaves. The pathogenicity of isolates in the *Epicoccum* genus is different; *E.dendrobii*, *E.italicum*, *E.jingdongense*, *E.mackenziei*, *E.oryzae*, *E.rosae* and *E.tobaicum* did not cause any disease symptoms, whereas *E.anhuiense*, *E.draconis*, *E.latusicollum*, *E.poaceicola* and *E.puerense* caused necrotic lesions on the tea plant leaves (Figs 11, 12). *Epicoccum* commonly display an endophytic lifestyle (Braga et al. 2018), so we speculated that the difference in pathogenicity may be due to the wound-inoculation method, which may result in the transition of some endophytes, such as *E.anhuiense* and *E.puerense* isolated from healthy leaves, to phytopathogens and the invasion of leaves from the artificial wounds. Therefore, the spray inoculation of healthy leaves in the future with conidia suspensions will help elucidate the pathogenic mechanism of all isolates. On the other hand, some *Epicoccum* species, such as *E.draconis*, *E.latusicollum* and *E.poaceicola* isolated from diseased leaves, were also reported as phytopathogens causing leaf spot on many plants, such as *Eugeniainvolucrata* (Bernardi et al. 2022), flowering cherry (Han et al. 2021), tobacco (Guo et al. 2020) and *Weigelaflorida* (Tian et al. 2021). Besides, *Epicoccum* species were mainly known as biocontrol agents against phytopathogens via inhibiting their growth and conidial germination (Braga et al. 2018). For example, *E.nigrum* limited the development of *Rhizoctoniasolani* in potato plants by growing along its hyphae and inducing lysis (Lahlali and Hijri 2010). In addition, *Epicoccum* species as endophytes can produce antifungal compounds, such as epicolactone that exhibits an inhibitory activity against *Remotididymellasolani*, epicoccamide D that induces morphogenesis and pigment formation in phytopathogenic fungus *Phomadestructiva* and flavipin that inhibits the growth of several fungal phytopathogens (Madrigal et al. 1991; Wangun et al. 2007; Fávaro et al. 2012; Talontsi et al. 2013). Therefore, endophytes isolated from tea plants, *E.dendrobii*, *E.italicum*, *E.jingdongense*, *E.mackenziei*, *E.oryzae*, *E.rosae* and *E.tobaicum*, may be beneficial species with biological control potential. Future studies could determine the inhibitory activity of these endophytes against the dominant pathogens in tea plants, such as *Colletotrichumcamelliae*, *C.fructicola*, *Didymellasegeticola*, *Exobasidiumvexans*, *Disculatheae-sinensis* and *Pestalotiopsistheae* and then identify the antifungal compounds.

The potential factors influencing the prevalence and pathogenicity of tested species in *Epicoccum* genus may be the different host-pathogen interaction patterns. Various infection strategies were deployed by pathogens to facilitate their own infection, such as secreting effectors, reprogramming the host transcriptome, rewiring host phytohormone signalling and disarming plant immune outputs (Wang et al. 2022). For *E.nigrum*, many strains secreted enzymes including amylases and proteases expected to participate mainly in the later stages of the infection (Ogórek et al. 2020). *Epicoccumsorghi* secreted polyglycine hydrolases to cleave the polyglycine linker of chitinases, antifungal proteins from *Zeamays* (Naumann et al. 2014; Naumann et al. 2017). To defend against diverse pathogens, plants have also evolved a robust innate immune system (Wang et al. 2022). Then, we speculated that *E.anhuiense*, *E.draconis*, *E.latusicollum*, *E.poaceicola* and *E.puerense* may adopt different infection strategies to invade tea plant (LJ43) leaves, resulting in the different outcome of host plant-pathogen interactions.

In summary, this study represents a comprehensive investigation of Didymellaceae family strains isolated from tea plant leaves of ten provinces in China. Combined with multi-locus (ITS, LSU, *RPB2* and *TUB2*) phylogenetic analysis and morphological characteristics, a total of 240 isolates were identified as 25 species of six genera, including 19 known species and six novel species. Amongst all isolates, *Didymellasegeticola* was the most dominant species. Pathogenicity analysis showed that their virulence varied. These results help us comprehend the diversity of Didymellaceae family in tea plants and provide a reference for disease management.

## Supplementary Material

XML Treatment for Didymella coffeae-arabicae

XML Treatment for Didymella pomorum

XML Treatment for Didymella segeticola

XML Treatment for Didymella sinensis

XML Treatment for Didymella yunnanensis

XML Treatment for Epicoccum anhuiense

XML Treatment for Epicoccum catenisporum

XML Treatment for Epicoccum dendrobii

XML Treatment for Epicoccum draconis

XML Treatment for Epicoccum italicum

XML Treatment for Epicoccum jingdongense

XML Treatment for Epicoccum latusicollum

XML Treatment for Epicoccum mackenziei

XML Treatment for Epicoccum oryzae

XML Treatment for Epicoccum poaceicola

XML Treatment for Epicoccum puerense

XML Treatment for Epicoccum rosae

XML Treatment for Epicoccum tobaicum

XML Treatment for Neoascochyta mortariensis

XML Treatment for Neoascochyta yunnanensis

XML Treatment for Neoascochyta zhejiangensis

XML Treatment for Paraboeremia litseae

XML Treatment for Remotididymella anemophila

XML Treatment for Stagonosporopsis caricae

## ﻿Additional information

### Author ORCIDs

Wuyun Lv  https://orcid.org/0000-0003-3781-0763

### Data availability

Sequence data from this study can be obtained from GenBank at https://www.ncbi.nlm.nih.gov/genbank/ with the accession numbers as listed in Suppl. material 1.

## Supplementary materials

Supplementary material 1 Isolates of the Didymellaceae family in this study and GenBank accession numbers of the generated sequences Data type docx https://binary.pensoft.net/file/1058581This dataset is made available under the Open Database License (http://opendatacommons.org/licenses/odbl/1.0/). The Open Database License (ODbL) is a license agreement intended to allow users to freely share, modify, and use this Dataset while maintaining this same freedom for others, provided that the original source and author(s) are credited.Yuchun Wang, Yiyi Tu, Xueling Chen, Hong Jiang, Hengze Ren, Qinhua Lu, Chaoling Wei, Wuyun Lv
