# Supplementary material for: ﻿Didymellaceae species associated with tea plant (Camelliasinensis) in China
Source: MycoKeys. 2024 May 29;105:217–51. doi: 10.3897/mycokeys.105.119536 (PMC11153891; doi:10.3897/mycokeys.105.119536)
Supplement: Supplementary material 1 — Isolates of the Didymellaceae family in this study and GenBank accession numbers of the generated sequences [file mycokeys-105-217-s001.docx]

**Table S1** Isolates of the *Didymellaceae* family in this study and GenBank accession numbers of the generated sequences.

| **Species** | **Collecting Location** | **Isolate /Culture** | **GenBank accessions** | | | |  |
| --- | --- | --- | --- | --- | --- | --- | --- |
|  |  | **collection** | **ITS** | ***rpb2*** | ***tub2*** | **LSU** |  |
| *Didymella coffeae-arabicae* | China, Yunnan, Puer | YCW1972 | OP647946 | OP854293 | / | / |  |
| *D. pomorum* | China, Yunnan | YCW196 | OP647945 | OP854292 | OP854550 | OP836938 |  |
| *D. segeticola* | China, Jiangsu, Yixing | YCW105 | OP647861 | OP854208 | OP854389 | OP836790 |  |
|  | China, Jiangsu, Yixing | YCW107 | OP647862 | OP854209 | OP854390 | OP836776 |  |
|  | China, Jiangsu, Yixing | YCW108 | OP647863 | OP854210 | OP854391 | OP836772 |  |
|  | China, Jiangsu, Yixing | YCW109 | OP647864 | OP854211 | OP854392 | OP836867 |  |
|  | China, Zhejiang, Hangzhou | YCW1096 | OP647865 | OP854212 | OP854393 | OP836791 |  |
|  | China, Zhejiang, Hangzhou | YCW1097 | OP647866 | OP854213 | OP854394 | OP836868 |  |
|  | China, Zhejiang, Hangzhou | YCW1098 | OP647867 | OP854214 | OP854505 | OP836770 |  |
|  | China, Jiangsu, Yixing | YCW11 | OP647868 | OP854215 | OP854395 | OP836792 |  |
|  | China, Jiangsu, Yixing | YCW110 | OP647869 | OP854216 | OP854396 | OP836793 |  |
|  | China, Zhejiang, Hangzhou | YCW1108 | OP647870 | OP854217 | OP854506 | OP836940 |  |
|  | China, Jiangsu, Wuxi | YCW1111 | OP647871 | OP854218 | OP854397 | OP836869 |  |
|  | China, Zhejiang, Hangzhou | YCW1112 | OP647872 | OP854219 | / | OP836922 |  |
|  | China, Zhejiang, Shaoxing | YCW1119 | OP647873 | OP854220 | / | OP836923 |  |

**Table S1** Continued.

| **Species** | **Collecting Location** | **Isolate /Culture** | **GenBank accessions** | | | |  |
| --- | --- | --- | --- | --- | --- | --- | --- |
|  |  | **collection** | **ITS** | ***rpb2*** | ***tub2*** | **LSU** |  |
|  | China, Jiangsu, Yixing | YCW112 | OP647874 | OP854221 | OP854398 | OP836794 |  |
|  | China, Zhejiang, Hangzhou | YCW1126 | OP647875 | OP854222 | OP854399 | OP836795 |  |
|  | China, Zhejiang, Hangzhou | YCW1127 | OP647876 | OP854223 | OP854400 | OP836796 |  |
|  | China, Zhejiang, Hangzhou | YCW1128 | OP647877 | OP854224 | OP854401 | OP836797 |  |
|  | China, Jiangsu, Yixing | YCW113 | OP647878 | OP854225 | OP854402 | OP836798 |  |
|  | China, Zhejiang, Hangzhou | YCW1130 | OP647879 | OP854226 | OP854403 | OP836870 |  |
|  | China, Zhejiang, Hangzhou | YCW1131 | OP647880 | OP854227 | OP854404 | OP836799 |  |
|  | China, Zhejiang, Hangzhou | YCW1132 | OP647881 | OP854228 | OP854405 | OP836800 |  |
|  | China, Zhejiang, Hangzhou | YCW1133 | OP647882 | OP854229 | OP854406 | OP836871 |  |
|  | China, Zhejiang, Hangzhou | YCW1134 | OP647883 | OP854230 | OP854407 | OP836801 |  |
|  | China, Zhejiang, Hangzhou | YCW1135 | OP647884 | OP854231 | OP854507 | OP836941 |  |
|  | China, Zhejiang, Hangzhou | YCW1136 | OP647885 | OP854232 | OP854408 | OP836802 |  |
|  | China, Zhejiang, Hangzhou | YCW1137 | OP647886 | OP854233 | OP854409 | OP836803 |  |
|  | China, Jiangsu, Yixing | YCW114 | OP647887 | OP854234 | OP854410 | OP836804 |  |
|  | China, Zhejiang, Hangzhou | YCW1140 | OP647888 | OP854235 | OP854411 | OP836881 |  |

**Table S1** Continued.

| **Species** | **Collecting Location** | **Isolate /Culture** | | **GenBank accessions** | | | |  |
| --- | --- | --- | --- | --- | --- | --- | --- | --- |
|  |  | **collection** | | **ITS** | ***rpb2*** | ***tub2*** | **LSU** |  |
|  | China, Jiangxi, Nanchang | YCW120 | | OP647889 | OP854236 | OP854525 | OP836912 |  |
|  | China, Jiangxi, Nanchang | YCW121 | | OP647890 | OP854237 | OP854526 | OP836913 |  |
|  | China, Zhejiang, Hangzhou | | YCW1211 | OP647891 | OP854238 | OP854412 | OP836773 |  |
|  | China, Jiangxi, Nanchang | YCW122 | | OP647892 | OP854239 | OP854413 | OP836891 |  |
|  | China, Jiangxi, Nanchang | YCW124 | | OP647893 | OP854240 | OP854527 | OP836914 |  |
|  | China, Zhejiang, Hangzhou | YCW1262 | | OP647894 | OP854241 | OP854414 | OP836872 |  |
|  | China, Zhejiang, Hangzhou | YCW1265 | | OP647895 | OP854242 | OP854415 | OP836805 |  |
|  | China, Zhejiang, Hangzhou | YCW1267 | | OP647896 | OP854243 | OP854416 | OP836806 |  |
|  | China, Zhejiang, Hangzhou | YCW1268 | | OP647897 | OP854244 | OP854417 | OP836807 |  |
|  | China, Zhejiang, Hangzhou | YCW1270 | | OP647898 | OP854245 | OP854418 | OP836808 |  |
|  | China, Zhejiang, Hangzhou | YCW1272 | | OP647899 | OP854246 | OP854419 | OP836809 |  |
|  | China, Zhejiang, Hangzhou | YCW1274 | | OP647900 | OP854247 | OP854420 | OP836810 |  |
|  | China, Zhejiang, Hangzhou | YCW1276 | | OP647901 | OP854248 | OP854421 | OP836811 |  |
|  | China, Zhejiang, Hangzhou | YCW1278 | | OP647902 | OP854249 | OP854422 | OP836873 |  |
|  | China, Zhejiang, Hangzhou | YCW1279 | | OP647903 | OP854250 | OP854423 | OP836812 |  |

**Table S1** Continued.

| **Species** | **Collecting Location** | **Isolate /Culture** | **GenBank accessions** | | | |  |
| --- | --- | --- | --- | --- | --- | --- | --- |
|  |  | **collection** | **ITS** | ***rpb2*** | ***tub2*** | **LSU** |  |
|  | China, Jiangxi, Nanchang | YCW128 | OP647904 | OP854251 | OP854424 | OP836874 |  |
|  | China, Zhejiang, Hangzhou | YCW1281 | OP647905 | OP854252 | OP854425 | OP836813 |  |
|  | China, Zhejiang, Hangzhou | YCW1283 | OP647906 | OP854253 | OP854426 | OP836814 |  |
|  | China, Zhejiang, Hangzhou | YCW1285 | OP647907 | OP854254 | OP854427 | OP836815 |  |
|  | China, Zhejiang, Hangzhou | YCW1287 | OP647908 | OP854255 | OP854428 | OP836816 |  |
|  | China, Zhejiang, Hangzhou | YCW1288 | OP647909 | OP854256 | OP854429 | OP836817 |  |
|  | China, Zhejiang, Hangzhou | YCW1289 | OP647910 | OP854257 | OP854430 | OP836875 |  |
|  | China, Jiangxi, Nanchang | YCW129 | OP647911 | OP854258 | / | OP836926 |  |
|  | China, Zhejiang, Hangzhou | YCW1290 | OP647912 | OP854259 | OP854431 | OP836818 |  |
|  | China, Zhejiang, Hangzhou | YCW1291 | OP647913 | OP854260 | OP854432 | OP836819 |  |
|  | China, Zhejiang, Hangzhou | YCW1292 | OP647914 | OP854261 | OP854433 | OP836820 |  |
|  | China, Zhejiang, Hangzhou | YCW1293 | OP647915 | OP854262 | OP854434 | OP836919 |  |
|  | China, Zhejiang, Hangzhou | YCW1294 | OP647916 | OP854263 | OP854435 | OP836821 |  |
|  | China, Zhejiang, Hangzhou | YCW1295 | OP647917 | OP854264 | OP854436 | OP836822 |  |
|  | China, Zhejiang, Hangzhou | YCW1296 | OP647918 | OP854265 | OP854508 | OP836921 |  |

**Table S1** Continued.

| **Species** | **Collecting Location** | **Isolate /Culture** | **GenBank accessions** | | | |  |
| --- | --- | --- | --- | --- | --- | --- | --- |
|  |  | **collection** | **ITS** | ***rpb2*** | ***tub2*** | **LSU** |  |
|  | China, Zhejiang, Hangzhou | YCW1297 | OP647919 | OP854266 | OP854437 | OP836823 |  |
|  | China, Zhejiang, Hangzhou | YCW1298 | OP647920 | OP854267 | OP854438 | OP836824 |  |
|  | China, Jiangxi, Nanchang | YCW131 | OP647921 | OP854268 | OP854439 | OP836876 |  |
|  | China, Jiangxi, Nanchang | YCW132 | OP647922 | OP854269 | OP854440 | OP836774 |  |
|  | China, Jiangxi, Nanchang | YCW133 | OP647923 | OP854270 | OP854441 | OP836777 |  |
|  | China, Jiangxi, Nanchang | YCW134 | OP647924 | OP854271 | OP854536 | OP836780 |  |
|  | China, Jiangxi, Nanchang | YCW135 | OP647925 | OP854272 | OP854537 | OP836781 |  |
|  | China, Yunnan | YCW1353 | OP647926 | OP854273 | OP854509 | OP836917 |  |
|  | China, Yunnan | YCW1358 | OP647927 | OP854274 | OP854510 | OP836918 |  |
|  | China, Jiangxi, Nanchang | YCW136 | OP647928 | OP854275 | OP854538 | OP836782 |  |
|  | China, Jiangxi, Nanchang | YCW138 | OP647929 | OP854276 | OP854539 | OP836783 |  |
|  | China, Jiangxi, Nanchang | YCW139 | OP647930 | OP854277 | OP854540 | OP836784 |  |
|  | China, Jiangxi, Nanchang | YCW140 | OP647931 | OP854278 | OP854541 | OP836935 |  |
|  | China, Jiangxi, Nanchang | YCW141 | OP647932 | OP854279 | OP854542 | OP836785 |  |
|  | China, Jiangxi, Nanchang | YCW146 | OP647933 | OP854280 | OP854543 | OP836788 |  |
|  | China, Sichuan, Chengdu | YCW150 | OP647934 | OP854281 | OP854511 | OP836896 |  |

**Table S1** Continued.

| **Species** | **Collecting Location** | **Isolate /Culture** | **GenBank accessions** | | | |  |
| --- | --- | --- | --- | --- | --- | --- | --- |
|  |  | **collection** | **ITS** | ***rpb2*** | ***tub2*** | **LSU** |  |
|  | China, Sichuan, Chengdu | YCW151 | OP647935 | OP854282 | OP854442 | OP836825 |  |
|  | China, Sichuan, Chengdu | YCW182 | OP647936 | OP854283 | OP854443 | OP836778 |  |
|  | China, Zhejiang, Lishui | YCW192 | OP647940 | OP854287 | OP854444 | OP836877 |  |
|  | China, Jiangsu, Yixing | YCW20 | OP647947 | OP854294 | OP854445 | OP836826 |  |
|  | China, Yunnan, Puer | YCW2007 | OP647948 | OP854295 | OP854512 | OP836934 |  |
|  | China, Yunnan | YCW205 | OP647949 | OP854296 | OP854528 | OP836907 |  |
|  | China, Yunnan | YCW208 | OP647950 | OP854297 | OP854446 | OP836827 |  |
|  | China, Yunnan | YCW211 | OP647952 | OP854299 | OP854447 | OP836828 |  |
|  | China, Yunnan | YCW215 | OP647954 | OP854301 | OP854448 | OP836829 |  |
|  | China, Yunnan | YCW216 | OP647955 | OP854302 | OP854544 | OP836786 |  |
|  | China, Zhejiang, Hangzhou | YCW2179 | OP647956 | OP854303 | / | OP836924 |  |
|  | China, Yunnan | YCW218 | OP647957 | OP854304 | / | OP836925 |  |
|  | China, Zhejiang, Hangzhou | YCW2180 | OP647958 | OP854305 | / | OP836928 |  |
|  | China, Zhejiang, Hangzhou | YCW2182 | OP647959 | OP854306 | / | OP836929 |  |
|  | China, Zhejiang, Hangzhou | YCW2183 | OP647960 | OP854307 | / | OP836930 |  |
|  | China, Zhejiang, Hangzhou | YCW2184 | OP647961 | OP854308 | / | OP836933 |  |
|  | China, Zhejiang, Hangzhou | YCW2186 | OP647962 | OP854309 | / | OP836931 |  |
|  | China, Zhejiang, Hangzhou | YCW2188 | OP647963 | OP854310 | / | OP836932 |  |

**Table S1** Continued.

| **Species** | **Collecting Location** | **Isolate /Culture** | **GenBank accessions** | | | |  |
| --- | --- | --- | --- | --- | --- | --- | --- |
|  |  | **collection** | **ITS** | ***rpb2*** | ***tub2*** | **LSU** |  |
|  | China, Jiangsu, Yixing | YCW22 | OP647964 | OP854311 | OP854449 | OP836830 |  |
|  | China, Yunnan | YCW221 | OP647965 | OP854312 | OP854450 | OP836831 |  |
|  | China, Yunnan | YCW222 | OP647966 | OP854313 | OP854451 | OP836832 |  |
|  | China, Yunnan | YCW226 | OP647967 | OP854314 | OP854545 | OP836787 |  |
|  | China, Yunnan | YCW227 | OP647968 | OP854315 | OP854546 | OP836789 |  |
|  | China, Yunnan | YCW228 | OP647969 | OP854316 | OP854452 | OP836833 |  |
|  | China, Guizhou, Tongren | YCW241 | OP647970 | OP854317 | OP854529 | OP836908 |  |
|  | China, Guizhou, Tongren | YCW247 | OP647971 | OP854318 | OP854453 | OP836882 |  |
|  | China, Guizhou, Tongren | YCW255 | OP647972 | OP854319 | OP854454 | OP836883 |  |
|  | China, Guizhou, Tongren | YCW256 | OP647973 | OP854320 | / | OP836937 |  |
|  | China, Jiangsu, Yixing | YCW26 | OP647974 | OP854321 | OP854455 | OP836884 |  |
|  | China, Guizhou, Tongren | YCW262 | OP647975 | OP854322 | OP854456 | OP836885 |  |
|  | China, Guizhou, Tongren | YCW265 | OP647976 | OP854323 | OP854457 | OP836834 |  |
|  | China, Jiangsu, Yixing | YCW30 | OP647977 | OP854324 | OP854530 | OP836769 |  |
|  | China, Anhui, Anqing | YCW312 | OP647978 | / | OP854458 | OP836936 |  |
|  | China, Anhui, Anqing | YCW316 | OP647979 | OP854325 | OP854459 | OP836779 |  |
|  | China, Hubei, Wuhan | YCW340 | OP647980 | OP854326 | OP854460 | OP836886 |  |

**Table S1** Continued.

| **Species** | **Collecting Location** | **Isolate /Culture** | **GenBank accessions** | | | |  |
| --- | --- | --- | --- | --- | --- | --- | --- |
|  |  | **collection** | **ITS** | ***rpb2*** | ***tub2*** | **LSU** |  |
|  | China, Hubei, Wuhan | YCW348 | OP647981 | OP854327 | OP854461 | OP836892 |  |
|  | China, Anhui, Huangshan | YCW368 | OP647982 | OP854328 | OP854513 | OP836916 |  |
|  | China, Anhui, Huangshan | YCW404 | OP647983 | OP854329 | OP854462 | OP836878 |  |
|  | China, Anhui, Huangshan | YCW406 | OP647984 | OP854330 | OP854463 | OP836835 |  |
|  | China, Anhui, Huangshan | YCW413 | OP647985 | OP854331 | OP854464 | OP836836 |  |
|  | China, Jiangsu, Yixing | YCW42 | OP647986 | OP854332 | OP854465 | OP836837 |  |
|  | China, Anhui, Huangshan | YCW435 | OP647987 | OP854333 | OP854466 | OP836838 |  |
|  | China, Anhui, Huangshan | YCW436 | OP647988 | OP854334 | OP854467 | OP836839 |  |
|  | China, Anhui, Huangshan | YCW438 | OP647989 | OP854335 | OP854531 | OP836909 |  |
|  | China, Anhui, Huangshan | YCW441 | OP647990 | OP854336 | OP854468 | OP836840 |  |
|  | China, Anhui, Huangshan | YCW443 | OP647991 | OP854337 | OP854532 | OP836915 |  |
|  | China, Anhui, Huangshan | YCW445 | OP647992 | OP854338 | OP854469 | OP836841 |  |
|  | China, Anhui, Huangshan | YCW446 | OP647993 | OP854339 | OP854470 | OP836842 |  |
|  | China, Anhui, Huangshan | YCW447 | OP647994 | OP854340 | / | OP836927 |  |
|  | China, Anhui, Huangshan | YCW448 | OP647995 | OP854341 | OP854471 | OP836843 |  |

**Table S1** Continued.

| **Species** | **Collecting Location** | **Isolate /Culture** | **GenBank accessions** | | | |  |
| --- | --- | --- | --- | --- | --- | --- | --- |
|  |  | **collection** | **ITS** | ***rpb2*** | ***tub2*** | **LSU** |  |
|  | China, Anhui, Huangshan | YCW452 | OP647996 | OP854342 | OP854472 | OP836879 |  |
|  | China, Anhui, Huangshan | YCW453 | OP647997 | OP854343 | OP854473 | OP836844 |  |
|  | China, Anhui, Huangshan | YCW454 | OP647998 | OP854344 | OP854474 | OP836845 |  |
|  | China, Anhui, Huangshan | YCW455 | OP647999 | OP854345 | OP854475 | OP836846 |  |
|  | China, Anhui, Huangshan | YCW456 | OP648000 | OP854346 | OP854476 | OP836847 |  |
|  | China, Anhui, Huangshan | YCW458 | OP648001 | OP854347 | OP854477 | OP836848 |  |
|  | China, Anhui, Huangshan | YCW461 | OP648002 | OP854348 | OP854533 | OP836910 |  |
|  | China, Anhui, Huangshan | YCW463 | OP648003 | OP854349 | OP854514 | OP836920 |  |
|  | China, Anhui, Huangshan | YCW464 | OP648004 | OP854350 | OP854534 | OP836911 |  |
|  | China, Jiangsu, Yixing | YCW48 | OP648005 | OP854351 | OP854478 | OP836849 |  |
|  | China, Anhui, Huangshan | YCW490 | OP648006 | OP854352 | OP854479 | OP836893 |  |
|  | China, Anhui, Huangshan | YCW493 | OP648007 | OP854353 | OP854480 | OP836887 |  |
|  | China, Anhui, Huangshan | YCW497 | OP648008 | OP854354 | OP854481 | OP836888 |  |
|  | China, Anhui, Huangshan | YCW504 | OP648009 | OP854355 | OP854482 | OP836880 |  |
|  | China, Jiangsu, Yixing | YCW52 | OP648010 | OP854356 | OP854483 | OP836850 |  |

**Table S1** Continued.

| **Species** | **Collecting Location** | **Isolate /Culture** | **GenBank accessions** | | | |  |
| --- | --- | --- | --- | --- | --- | --- | --- |
|  |  | **collection** | **ITS** | ***rpb2*** | ***tub2*** | **LSU** |  |
|  | China, Anhui, Huangshan | YCW523 | OP648011 | OP854357 | OP854484 | OP836894 |  |
|  | China, Jiangsu, Yixing | YCW53 | OP648012 | OP854358 | OP854485 | OP836851 |  |
|  | China, Anhui, Huangshan | YCW536 | OP648013 | OP854359 | OP854486 | OP836852 |  |
|  | China, Hubei, Wuhan | YCW541 | OP648014 | OP854360 | OP854515 | OP836771 |  |
|  | China, Hubei, Wuhan | YCW546 | OP648015 | OP854361 | OP854487 | OP836889 |  |
|  | China, Hubei, Wuhan | YCW547 | OP648016 | OP854362 | OP854488 | OP836895 |  |
|  | China, Jiangsu, Yixing | YCW64 | OP648017 | OP854363 | OP854489 | OP836853 |  |
|  | China, Jiangsu, Yixing | YCW67 | OP648018 | OP854364 | OP854490 | OP836854 |  |
|  | China, Jiangsu, Yixing | YCW68 | OP648019 | OP854365 | OP854491 | OP836855 |  |
|  | China, Guangdong, Guangzhou | YCW695 | OP648020 | OP854366 | OP854492 | OP836775 |  |
|  | China, Jiangsu, Yixing | YCW7 | OP648021 | OP854367 | OP854493 | OP836856 |  |
|  | China, Jiangsu, Yixing | YCW70 | OP648022 | OP854368 | OP854494 | OP836857 |  |
|  | China, Jiangsu, Yixing | YCW71 | OP648023 | OP854369 | OP854495 | OP836858 |  |
|  | China, Jiangsu, Yixing | YCW73 | OP648024 | OP854370 | OP854516 | OP836897 |  |
|  | China, Jiangsu, Yixing | YCW75 | OP648025 | OP854371 | OP854517 | OP836898 |  |

**Table S1** Continued.

| **Species** | **Collecting Location** | **Isolate /Culture** | **GenBank accessions** | | | |  |
| --- | --- | --- | --- | --- | --- | --- | --- |
|  |  | **collection** | **ITS** | ***rpb2*** | ***tub2*** | **LSU** |  |
|  | China, Jiangsu, Yixing | YCW77 | OP648026 | OP854372 | OP854518 | OP836899 |  |
|  | China, Jiangsu, Yixing | YCW8 | OP648027 | OP854373 | OP854496 | OP836859 |  |
|  | China, Jiangsu, Yixing | YCW83 | OP648028 | OP854374 | OP854497 | OP836860 |  |
|  | China, Jiangsu, Yixing | YCW85 | OP648029 | OP854375 | OP854498 | OP836861 |  |
|  | China, Jiangsu, Yixing | YCW89 | OP648030 | OP854376 | OP854499 | OP836862 |  |
|  | China, Jiangsu, Yixing | YCW90 | OP648031 | OP854377 | OP854500 | OP836863 |  |
|  | China, Sichuan, Guangyuan | YCW902 | OP648032 | OP854378 | OP854501 | OP836890 |  |
|  | China, Zhejiang, Hangzhou | YCW907 | OP648033 | OP854379 | OP854502 | OP836864 |  |
|  | China,Anhui, Anqing | YCW959 | OP648034 | OP854380 | OP854503 | OP836865 |  |
|  | China,Anhui, Anqing | YCW960 | OP648035 | OP854381 | OP854519 | OP836900 |  |
|  | China,Anhui, Anqing | YCW963 | OP648036 | OP854382 | OP854520 | OP836901 |  |
|  | China,Anhui, Anqing | YCW966 | OP648037 | OP854383 | OP854521 | OP836902 |  |
|  | China,Anhui, Anqing | YCW968 | OP648038 | OP854384 | OP854522 | OP836903 |  |
|  | China,Anhui, Anqing | YCW969 | OP648039 | OP854385 | OP854523 | OP836904 |  |
|  | China,Anhui, Anqing | YCW978 | OP648040 | OP854386 | OP854535 | OP836906 |  |

**Table S1** Continued.

| **Species** | **Collecting Location** | **Isolate /Culture** | **GenBank accessions** | | | |  |
| --- | --- | --- | --- | --- | --- | --- | --- |
|  |  | **collection** | **ITS** | ***rpb2*** | ***tub2*** | **LSU** |  |
|  | China,Anhui, Anqing | YCW980 | OP648041 | OP854387 | OP854504 | OP836866 |  |
|  | China,Anhui, Anqing | YCW987 | OP648042 | OP854388 | OP854524 | OP836905 |  |
| *D. sinensis* | China, Yunnan, Puer | YCW1884 | OP647937 | OP854284 | OP854547 | / |  |
|  | China, Yunnan, Puer | YCW1906 | OP647938 | OP854285 | OP854548 | / |  |
|  | China, Yunnan, Puer | YCW1934 | OP647941 | OP854288 | / | / |  |
|  | China, Yunnan, Puer | YCW1946 | OP647942 | OP854289 | / | / |  |
|  | China, Yunnan, Puer | YCW1950 | OP647943 | OP854290 | OP854549 | / |  |
|  | China, Yunnan, Puer | YCW1951 | OP647944 | OP854291 | / | / |  |
|  | China, Yunnan, Puer | YCW2095 | OP647951 | OP854298 | / | / |  |
|  | China, Yunnan, Puer | YCW2118 | OP647953 | OP854300 | / | / |  |
| *D. yunnanensis* | China, Yunnan, Puer | **CGMCC 3.24241; YCW1909** | OP647939 | OP854286 | OP854551 | OP836939 |  |
| *Epicoccum anhuiense* | China, Yunnan, Puer | YCW1825 | OP648053 | OP716594 | OP854566 | OP837086 |  |
|  | China, Yunnan, Puer | CGMCC 3.24246; YCW1829 | OP648054 | OP716595 | OP854567 | OP837087 |  |
|  | China, Yunnan, Puer | YCW2011 | OP648055 | OP716596 | OP854568 | OP837088 |  |

**Table S1** Continued.

| **Species** | **Collecting Location** | **Isolate /Culture** | **GenBank accessions** | | | |  |
| --- | --- | --- | --- | --- | --- | --- | --- |
|  |  | **collection** | **ITS** | ***rpb2*** | ***tub2*** | **LSU** |  |
|  | China, Yunnan, Puer | YCW2035 | OP648056 | OP716597 | OP854569 | OP837089 |  |
|  | China, Yunnan, Puer | YCW1929 | OP648057 | OP716598 | / | OP837090 |  |
|  | China, Anhui, Anqing | **CGMCC 3.24242; YCW961** | OP648058 | OP716599 | OP854570 | OP837091 |  |
| *E. catenisporum* | China, Jiangxi, Nanchang | YCW142 | OP648062 | OP716603 | OP854571 | OP837092 |  |
| *E. dendrobii* | China, Yunnan, Puer | YCW1866 | OP648071 | OP716612 | / | / |  |
|  | China, Yunnan | YCW201 | OP648076 | OP716617 | OP854581 | OP837101 |  |
|  | China, Yunnan, Puer | YCW2101 | OP648084 | OP716625 | / | / |  |
| *E. draconis* | China, Jiangsu, Yixing | YCW101 | OP648043 | OP716584 | OP854557 | OP837077 |  |
|  | China, Zhejiang, Lishui | YCW187 | OP648044 | OP716585 | OP854558 | OP837078 |  |
| *E. italicum* | China, Yunnan, Puer | YCW2005 | OP648088 | OP716629 | / | / |  |
| *E. jingdongense* | China, Yunnan, Puer | **CGMCC 3.24247; YCW1868** | OP648072 | OP716613 | OP854577 | OP837098 |  |
|  | China, Yunnan, Puer | CGMCC 3.24248; YCW1937 | OP648073 | OP716614 | OP854578 | OP837099 |  |
|  | China, Yunnan, Puer | YCW1938 | OP648074 | OP716615 | OP854579 | OP837100 |  |
|  | China, Yunnan, Puer | YCW2024 | OP648075 | OP716616 | OP854580 | / |  |

**Table S1** Continued.

| **Species** | **Collecting Location** | **Isolate /Culture** | **GenBank accessions** | | | |  |
| --- | --- | --- | --- | --- | --- | --- | --- |
|  |  | **collection** | **ITS** | ***rpb2*** | ***tub2*** | **LSU** |  |
|  | China, Yunnan, Puer | YCW1989 | OP648082 | OP716623 | OP854587 | OP837107 |  |
|  | China, Yunnan, Puer | YCW2088 | OP648083 | OP716624 | / | OP837108 |  |
| *E. latusicollum* | China, Yunnan, Puer | YCW1921 | OP648059 | OP716600 | / | / |  |
|  | China, Yunnan, Puer | YCW1968 | OP648060 | OP716601 | / | / |  |
|  | China, Yunnan, Puer | YCW2015 | OP648061 | OP716602 | / | / |  |
| *E. mackenziei* | China, Yunnan, Puer | CGMCC 3.24244; YCW1965 | OP648085 | OP716626 | OP854588 | OP837109 |  |
|  | China, Yunnan, Puer | CGMCC 3.24245; YCW1967 | OP648086 | OP716627 | / | OP837110 |  |
| *E. oryzae* | China, Yunnan, Puer | YCW2010 | OP648087 | OP716628 | / | OP837111 |  |
| *E. poaceicola* | China, Yunnan, Puer | YCW1804 | OP648046 | OP716587 | OP854559 | OP837079 |  |
|  | China, Yunnan, Puer | YCW1948 | OP648047 | OP716588 | OP854560 | OP837080 |  |
|  | China, Yunnan, Puer | YCW1966 | OP648048 | OP716589 | OP854561 | OP837081 |  |
|  | China, Yunnan, Puer | YCW2023 | OP648049 | OP716590 | OP854562 | OP837082 |  |
|  | China, Yunnan, Puer | YCW2115 | OP648050 | OP716591 | OP854563 | OP837083 |  |
|  | China, Yunnan, Puer | YCW1841 | OP648051 | OP716592 | OP854564 | OP837084 |  |

**Table S1** Continued.

| **Species** | **Collecting Location** | **Isolate /Culture** | **GenBank accessions** | | | |  |
| --- | --- | --- | --- | --- | --- | --- | --- |
|  |  | **collection** | **ITS** | ***rpb2*** | ***tub2*** | **LSU** |  |
|  | China, Yunnan, Puer | YCW2001 | OP648052 | OP716593 | OP854565 | OP837085 |  |
| *E. puerense* | China, Yunnan, Puer | YCW1876 | OP648077 | OP716618 | OP854582 | OP837102 |  |
|  | China, Yunnan | CGMCC 3.24243; YCW224 | OP648078 | OP716619 | OP854583 | OP837103 |  |
|  | China, Yunnan | YCW225 | OP648079 | OP716620 | OP854584 | OP837104 |  |
|  | China, Yunnan, Puer | **CGMCC 3.24249; YCW2117** | OP648080 | OP716621 | OP854585 | OP837105 |  |
|  | China, Yunnan | YCW202 | OP648081 | OP716622 | OP854586 | OP837106 |  |
| *E. rosae* | China, Hubei, Wuhan | YCW331 | OP648066 | OP716607 | OP854572 | OP837093 |  |
|  | China, Anhui, Huangshan | YCW472 | OP648067 | OP716608 | OP854573 | OP837094 |  |
|  | China, Jiangsu, Yixing | YCW46 | OP648068 | OP716609 | OP854574 | OP837095 |  |
| *E. tobaicum* | China, Zhejiang, Hangzhou | YCW1103 | OP648063 | OP716604 | / | / |  |
|  | China, Hubei, Wuhan | YCW333 | OP648064 | OP716605 | / | / |  |
|  | China, Henan, Xinyang | YCW828 | OP648065 | OP716606 | / | / |  |
|  | China, Hubei, Wuhan | YCW336 | OP648069 | OP716610 | OP854575 | OP837096 |  |
|  | China, Anhui, Huangshan | YCW372 | OP648070 | OP716611 | OP854576 | OP837097 |  |
| *E. sorghinum* | China, Zhejiang, Hangzhou | YCW1338 | OP648045 | OP716586 | / | / |  |

**Table S1** Continued.

| **Species** | **Collecting Location** | **Isolate /Culture** | **GenBank accessions** | | | |  |
| --- | --- | --- | --- | --- | --- | --- | --- |
|  |  | **collection** | **ITS** | ***rpb2*** | ***tub2*** | **LSU** |  |
| *Neoascochyta mortariensis* | China, Zhejiang, Hangzhou | CGMCC 3.24251; YCW1346 | OP648089 | OP716579 | OP854552 | OP837279 |  |
| *Neoascochyta* sp. | China, Zhejiang, Hangzhou | YCW1124 | OP648093 | OP716583 | OP854556 | OP837283 |  |
| *N. yunnanensis* | China, Yunnan, Puer | **CGMCC 3.24253; YCW1883** | OP648090 | OP716580 | OP854553 | OP837280 |  |
| *N. zhejiangensis* | China, Yunnan | CGMCC 3.24251; YCW1361 | OP648091 | OP716581 | OP854554 | OP837281 |  |
|  | China, Zhejiang, Hangzhou | **CGMCC 3.24253; YCW1107** | OP648092 | OP716582 | OP854555 | OP837282 |  |
| *Paraboeremia litseae* | China, Yunnan | YCW1356 | OP648095 | OP716577 | OP854589 | OP837286 |  |
|  | China, Yunnan | YCW1363 | OP648096 | OP716578 | OP854590 | OP837287 |  |
| *Remotididymella anemophila* | China, Anhui, Huangshan | YCW499 | OP648106 | OP716574 | OP854591 | OP837290 |  |
|  | China, Zhejiang, Hangzhou | YCW1118 | OP648107 | OP716575 | OP854592 | OP837291 |  |
|  | China, Anhui, Huangshan | YCW434 | OP648108 | OP716576 | OP854593 | / |  |
| *Stagonosporopsis caricae* | China, Yunnan, Puer | YCW1928 | OP648100 | / | OP854594 | OP837293 |  |
|  | China, Yunnan, Puer | YCW1977 | OP648101 | / | OP854595 | OP837294 |  |

Ex-type strains are emphasized in bold.
